# Clinically translatable cytokine delivery platform for eradication of intraperitoneal tumors

**DOI:** 10.1126/sciadv.abm1032

**Published:** 2022-03-02

**Authors:** Amanda M. Nash, Maria I. Jarvis, Samira Aghlara-Fotovat, Sudip Mukherjee, Andrea Hernandez, Andrew D. Hecht, Peter D. Rios, Sofia Ghani, Ira Joshi, Douglas Isa, Yufei Cui, Shirin Nouraein, Jared Z. Lee, Chunyu Xu, David Y. Zhang, Rahul A. Sheth, Weiyi Peng, Jose Oberholzer, Oleg A. Igoshin, Amir A. Jazaeri, Omid Veiseh

**Affiliations:** 1 Department of Bioengineering, Rice University, Houston, TX, USA.; 2 CellTrans Inc., Chicago, IL, USA.; 3 Department of Chemistry, Rice University, Houston, TX, USA.; 4 Department of Biology and Biochemistry, The University of Houston, Houston, TX, USA.; 5 Department of Interventional Radiology, The University of Texas MD Anderson Cancer Center, Houston, TX, USA.; 6 Department of Surgery, University of Virginia, Charlottesville, VA, USA.; 7 Department of Gynecologic Oncology and Reproductive Medicine, Division of Surgery, The University of Texas MD Anderson Cancer Center, Houston, TX, USA.

## Abstract

Proinflammatory cytokines have been approved by the Food and Drug Administration for the treatment of metastatic melanoma and renal carcinoma. However, effective cytokine therapy requires high-dose infusions that can result in antidrug antibodies and/or systemic side effects that limit long-term benefits. To overcome these limitations, we developed a clinically translatable cytokine delivery platform composed of polymer-encapsulated human ARPE-19 (RPE) cells that produce natural cytokines. Tumor-adjacent administration of these capsules demonstrated predictable dose modulation with spatial and temporal control and enabled peritoneal cancer immunotherapy without systemic toxicities. Interleukin-2 (IL2)–producing cytokine factory treatment eradicated peritoneal tumors in ovarian and colorectal mouse models. Furthermore, computational pharmacokinetic modeling predicts clinical translatability to humans. Notably, this platform elicited T cell responses in NHPs, consistent with reported biomarkers of treatment efficacy without toxicity. Combined, our findings demonstrate the safety and efficacy of IL2 cytokine factories in preclinical animal models and provide rationale for future clinical testing in humans.

## Teaser

Polymer-encased cells offer localized delivery and spatial/temporal dosing control of cytokines for cancer immunotherapy.

## INTRODUCTION

Cytokines are soluble molecular messengers that activate and propagate disease-fighting immune cascades in the body in response to stimuli from antigen-presenting cells (*1*–*3*). In cancer immunotherapy, exogenous cytokines initiate immune system activation to optimize the magnitude and nature of the acquired antitumor immune response (*4*). Many proinflammatory cytokines including interleukin-2 (IL2), IL7, IL12, IL15, and interferon-γ have shown promise for antitumor efficacy in clinical trials (*1*, *5*, *6*). IL2 is of particular interest for immuno-oncology because of the critical role it plays in the regulation of immune cells such as T cells (*7*–*9*). High-dose bolus administration of recombinant IL2 treatment has been Food and Drug Administration (FDA)–approved for use in melanoma and renal cancers under the trade names Aldesleukin or Proleukin since 1992 (*8*, *10*). However, the half-life of IL2 in the blood is on the order of minutes, and high-dose infusion regimens are needed to elicit substantial tumor reduction in patients with cancer (*8*, *11*, *12*). Unfortunately, various off-target effects and toxicities are experienced by many patients, hindering the widespread adoption of this treatment (*13*, *14*). Among the many severe side effects, vascular leak syndrome can lead to fluid accumulation and tissue damage in major organs such as the liver, heart, and kidney (*15*, *16*). As a result, patients are often forced to be taken off infusions and/or removed from clinical trials. In addition, development of antidrug antibodies in response to repeat administration of high-dose recombinant IL2 has limited the efficacy and feasibility of this treatment option (*17*, *18*).

Native IL2, which is naturally produced by cells in the body, is structurally similar to recombinant IL2, but it diverges in bioactivity and functionality and has not been commercialized because of the inherent instability of the molecule (*19*). Instead, in attempts to improve recombinant IL2 safety and pharmacokinetics, researchers have taken advantage of advances in protein engineering and controlled release formulations (*20*). Recombinant cytokines with stability-enhancing modifications including various IL2 analogs, IL2 conjugates, and IL2 fusion proteins have been created (*12*, *21*–*25*). In addition, controlled release systems that can deliver small amounts of cytokines over time have been designed to address dosing limitations (*26*). A hyperstable de novo mimic of IL2 with reduced affinity to α chain subunit of the IL2 receptor was also created to limit its pleotropic bioactivity (*27*). More recently, injectable mRNA-based formulations have been developed to enable IL2 production directly from cancer cells, but these formulations do not allow for control over dosing and duration of therapy, which is especially problematic given the pleotropic nature of cytokines such as IL2 (*28*). Overall, while many of these approaches have demonstrated promising results, especially in combination with other antitumor agents, an approach to simultaneously account for the short lifetime, maintain the full biological activity, and limit systemic toxicity of IL2 remains elusive.

In contrast to conventional intravenous and subcutaneous routes of administration (*29*), local delivery of IL2 has the potential to resolve the activity-toxicity trade-off. For cancers with frequent metastasis to the peritoneal cavity, intraperitoneal (IP) administration is a promising possibility. The peritoneal cavity is separated from the systemic circulation by the peritoneal wall, and therefore, infusion of IL2 directly into this compartmentalized space could increase the therapeutic potential of the treatment while simultaneously reducing the off-target effects associated with intravenous and subcutaneous routes of administration (*29*). A phase 2 clinical trial of patients with ovarian cancer with platinum-resistant tumors was conducted with weekly IP administered recombinant IL2 (*30*). In this study among the 24 patients, an overall response rate of 25.0% was observed (four complete and two partial). Unlike intravenously administered IL2 (*14*, *30*), IP administration was associated with a much more tolerable toxicity profile (*30*). Reduction of the side effects was mainly due to a ~100-fold difference between the local concentration of IL2 in the IP cavity and that in blood. Moreover, IL2 remained in the IP space for many days after administration, demonstrating its stability in that environment. These results suggested that local administration of IL2 could help reduce the clinically relevant dose needed (*31*). However, the large infusion volumes (>1 liter/week) needed for IP IL2 immunotherapy can disrupt the IP diffusion barrier and natural compartmentalization and thus sacrifice the critical concentration differential between the local and systemic spaces.

Here, we develop living cytokine factories, a platform for localized delivery of natural, cell-generated cytokines with spatial and temporal control over dosing. We assess the ability of this system to reduce IP tumor burden in murine models of ovarian (*32*) and colorectal cancer (*33*), and we evaluate the safety and clinical translatability of our approach through pharmacokinetic (PK) modeling and PK and pharmacodynamic (PK/PD) measurements along with safety profile assessments in nonhuman primates (*34*).

## RESULTS

### Design and fabrication of cytokine factories

**Fig. 1.**
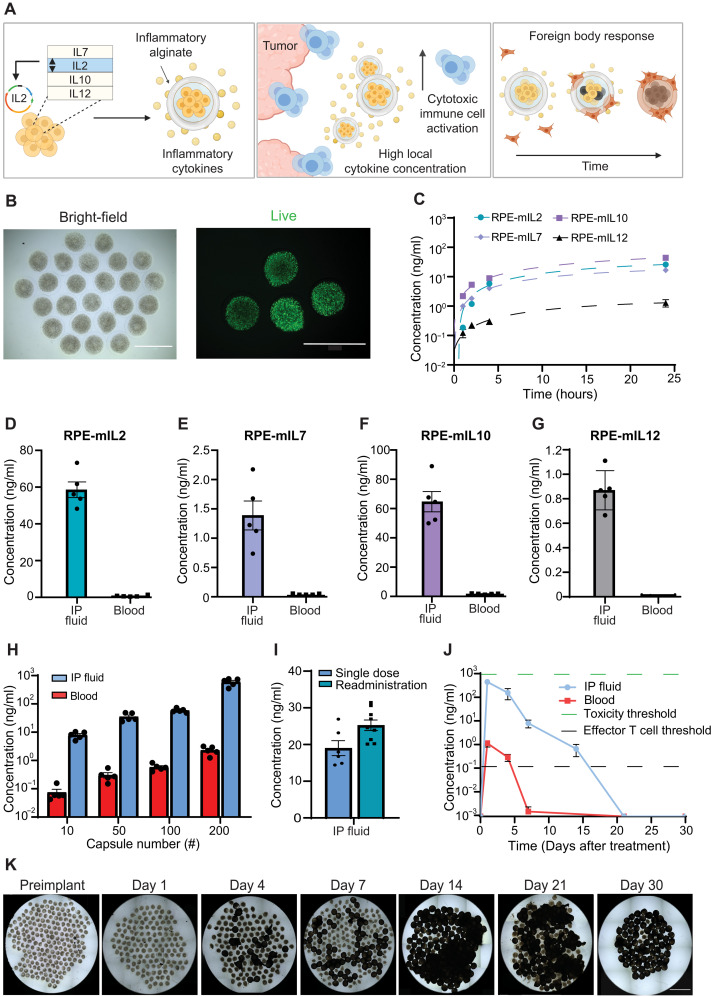
Development of encapsulated cells as cytokine factories with tuned in vivo pharmacokinetics. (**A**) Schematic of implantable cytokine factory production and in vivo immune responses. (**B**) Capsules containing RPE cells [bright-field and fluorescence (green fluorescent protein), 2×]. Scale bars, 2 mm. (**C**) Enzyme-linked immunosorbent assay (ELISA) measurements of mouse IL2 (mIL2), mIL7, mIL10, and mIL12 (*n* = 4, means ± SEM) from capsules after 1, 2, 4, and 24 hours. Dashed lines represent quadratic polynomial fit. (**D** to **G**) In vivo cytokine expression levels (ng/ml) measured 24 hours after IP implantation (100 capsules, *n* = 5). *P* values (*P* < 0.0001 for all groups) were determined using multiple *t* tests. (D) is representative of three independent experiments. (**H**) In vivo mIL2 concentrations as a function of capsule dose (*n* = 5) 24 hours after implantation. Data are representative of two independent experiments. (**I**) mIL2 concentration in IP fluid 24 hours after capsule implantation. Data are representative of two independent experiments. (**J**) In vivo mIL2 concentrations as a function of time with a fixed dose of 200 capsules (*n* = 5, means ± SEM). Effector T cell and toxicity thresholds were determined from reported literature values. Data are representative of two independent experiments. Capsules collected from PK studies were imaged (**K**) using bright-field microscopy (2×) to qualitatively monitor PFO on the surface over time. Single field of view (2×) images with overlapping fields were stitched to create a mosaic.

Our proinflammatory cytokine delivery system consisted of human retinal pigmented epithelial (RPE) cells that were engineered to stably express a proinflammatory cytokine of choice (Fig. 1A) using the PiggyBac transposon system. RPE cells were chosen because they are nontumorigenic (*35*), display contact inhibition (*36*), are amenable to genetic modification (*35*), have been previously used in human trials for the delivery of therapeutics, and have been shown to be safe (*37*–*39*). Interchanging the appropriate sequence for the gene of interest while maintaining the optimized backbone allows for rapid prototype development. Engineered cells were encapsulated within alginate-based microparticles (capsules) using a coaxial needle and cross-linking bath. We found that the cells remain viable after encapsulation (Fig. 1B), do not divide within the capsules (fig. S1), produce the cytokine of interest (fig. S2), and persist longer in vivo than unencapsulated cells (fig. S3, A and B). These results demonstrate that encapsulation was well tolerated by the cells. Following encapsulation, the cytokine factories are referred to as RPE-mIL2, RPE-mIL7, RPE-mIL10, RPE-mIL12, or RPE-hIL2, depending on which cytokine they were engineered to produce.

To evaluate the production kinetics of our engineered cells in vitro, cytokine production from a series of individual capsules was tracked for 24 hours (Fig. 1C). In vitro cytokine production increased at the early time points (1, 2, and 4 hours), but between 4 and 24 hours, a balance between cytokine production and degradation was observed. However, our approach incorporates living cells that will continue to replenish any degraded cytokines over time.

For human translatability, RPE-hIL2 was also created and human IL2 (hIL2) production was tracked over time (fig. S4). The production kinetics of the hIL2 mirrored the results seen with the mouse cytokines and suggested that our plug-and-play cell engineering approach was also compatible with human cytokines. We next tested the ability of our cell-produced cytokines to bind to IL2 receptors using a commercial HEK-Blue IL2 reporter cell line. Figure S5 shows that RPE-hIL2 binds to IL2 receptors with a similar affinity as the clinical hIL2 standard between 10^−3^ and 10^2^ ng/ml of hIL2. These results highlighted that the cytokines produced by the RPE-hIL2–produced proteins were properly processed and folded (*40*).

### Cytokine factories allow spatial and temporal control of cytokine delivery in mice

The IP fluid is separated from the systemic circulation (bloodstream) by the peritoneal wall. Thus, we hypothesized that capsules implanted within the IP cavity would create high cytokine concentrations within the IP cavity while maintaining low concentrations in the blood. To validate this hypothesis, we implanted 100 capsules in the IP cavity of C57BL/6 mice for 24 hours and quantified protein levels in the IP fluid and blood using enzyme-linked immunosorbent assay (ELISA). For each of the tested cytokines, the local concentration (IP space) was at least 30× higher than the systemic concentration. These results demonstrate the ability of our platform to deliver native cytokines in vivo and create a high local concentration of cytokines without excessive accumulation in the blood (Fig. 1, D to G). Considering the success and substantial therapeutic potential of IL2 described above, we focused further studies on the RPE-mIL2 and RPE-hIL2 cytokine factories.

Given the pleiotropic nature of IL2 bioactivity (*8*), control of dosing is critical for robust and efficacious immunotherapy. To test the ability to tune the local concentration of mouse IL2 (mIL2) delivered in vivo, we varied the number of capsules within a given dose. Four doses of RPE-mIL2 capsules—10 (1.3 μg mIL2/day), 50 (6.5 μg mIL2/day), 100 (13.1 μg mIL2/day), and 200 (26.2 μg mIL2/day)—were implanted in the IP cavity, and the in vivo concentration of mIL2 was assessed after 24 hours. We determined that the local IP concentration of mIL2 increased with the number of capsules delivered and that the mIL2 levels in the IP fluid were consistently more than 100× higher than in the blood (Fig. 1H). Notably, expression was consistent across mice in each group, highlighting our platform’s ability to tightly control cytokine production in vivo by adjusting the number of capsules in each dose.

We next evaluated whether additional rounds of capsule administration would be feasible. This key feature would allow clinicians the flexibility to dose multiple times, if needed, to achieve tumor regression or test a particular patient’s ability to tolerate the therapy. To evaluate the feasibility of capsule redosing, we administered 25 RPE-mIL2 capsules for 30 days. Then, an additional 25 capsules were administered and compared to control mice (one 25-capsule dose). As expected, the mIL2 concentrations in the IP fluid of mice from both single- and double-administration groups were similar, demonstrating successful redosing of our cytokine delivery platform without inducing dose-altering antidrug (mIL2) antibody formation (Fig. 1I).

In addition to tunable dosing, temporal control to ensure the production turns off after completion of therapy is also necessary. The natural foreign body response (FBR) to immunostimulatory SLG20 hydrogel microparticles has been previously shown to cause pericapsular fibrotic overgrowth (PFO) on the capsule surface, a subsequent decrease in pore size, and eventual cell death when oxygen and nutrients cease to diffuse inside (*41*). We found that the mIL2 concentration peaked 24 hours after implantation without crossing the toxicity threshold and remained well above the threshold for effector T cell binding (*42*) for at least 14 days before the capsules became coated by the host immune system (Fig. 1J). Imaging of the explanted capsules showed that the percentage of fibrosed capsules was inversely correlated to the mIL2 concentration in the blood and IP fluid (Fig. 1K). These data highlight that administration of our hydrogel platform is safe and predictable. No significant deviations from starting body weight were observed following administration of RPE-mIL2 treatment in this study.

### RPE-mIL2 eradicates tumor burden in a mouse model of advanced ovarian cancer

**Fig. 2.**
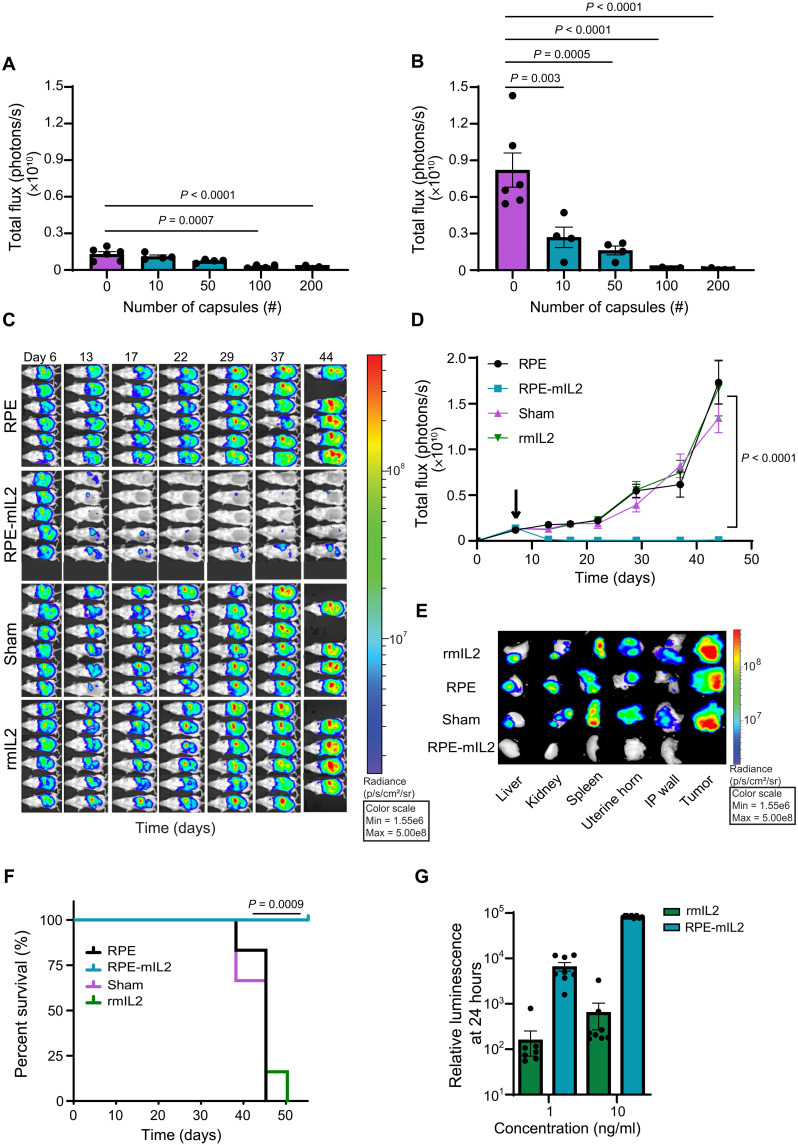
RPE-mIL2 treatment has a significant effect in reducing tumor burden over time in ovarian cancer mouse models. Luminescent images tracking tumor burden as a function of RPE-mIL2 dose at (**A**) day 6 and (**B**) day 30 after treatment (means ± SEM). *P* values were determined using one-way analysis of variance (ANOVA) with the Holm-Sidak method for multiple comparisons. Data are from one dedicated experiment. (**C**) Luminescent images of mice (*n* = 5 to 6) over time. Data are representative of two independent experiments. (**D**) Tumor burden (*n* = 5 to 6) represented by total flux (photons/s) plotted over time. The black arrow indicates RPE-mIL2 administration (7 days after injection; means ± SEM). *P* values were determined by two-way ANOVA, using the Holm-Sidak method for multiple comparisons. (**E**) Explanted organs and tumors collected from the IP space. (C) and (E) were collected using an f-stop of 1.2, 15 s of exposure, and a field of view at 24 and 12, respectively. (**F**) Survival curves (*n* = 5 to 6) depicted as percent survival over time in days after tumor injection. Comparison of survival curves was done using the log-rank, Mantel-Cox test. (**G**) Relative luminescence of 1 × 10^4^ isolated murine T cells 24 hours (*n* = 8) after treatment with rmIL2 or mIL2 secreted from RPE-mIL2. *P* values were determined to be *P* < 0.001 using two-way ANOVA, using the Holm-Sidak method for multiple comparisons. Data are representative of two independent experiments.

To test the efficacy of our platform in a mouse model of advanced ovarian cancer, we treated IP ID8-Fluc tumor-bearing mice with various doses of RPE-mIL2. After only 6 days of treatment, mice with 100 or 200 capsules exhibited a reduction in tumor burden that was 3.3× and 7.5×, respectively, greater than sham mice (Fig. 2A). After 30 days, there was a significant reduction in tumor burden across all groups that received RPE-mIL2 capsules (Fig. 2B). When compared to sham mice, mice treated with 10, 50, 100, or 200 RPE-mIL2 showed 3.1×, 5×, 53×, and 147× less tumor burden, respectively. These results were independently repeated two times, and a total of 20 mice were treated with RPE-mIL2. A total of 100% of these animals showed significant tumor burden reduction at day 30 when compared to sham mice. Luminescent images for all mice at both 6 and 30 days after treatment are displayed in fig. S6. These data highlight the therapeutic effect of tumor-adjacent delivery of RPE-mIL2.

To evaluate the therapeutic efficacy of our delivery platform more in comparison to recombinant mIL2 (rmIL2) or the alginate biomaterial system alone, we performed an in vivo survival study. The ovarian cancer mouse model was established as described in Materials and Methods. The group stratification can be seen in fig. S7 (A and B). The total flux from all mice that received RPE-mIL2 dropped in less than 1 week of treatment and remained below initial values for the duration of the study (Fig. 2, C and D). No significant deviations from the starting body weight were observed following administration of RPE-mIL2 treatment in this study. To evaluate tumor metastasis, we euthanized animals, and the organs (liver, kidney, spleen, uterine horn, and IP wall) and tumor specimens were collected and imaged ex vivo. RPE-mIL2–treated mice were tumor free across all major organs, and no tumor mass was found within the IP space (Fig. 2E). The mice in each of the control groups displayed intense tumor burden in each of the organs mentioned and showed large tumor masses in the IP space as well as hemoperitoneum and severe organ discoloration. All RPE-mIL2 mice displayed tumor reduction by at least 90% after 15 days. These mice remained tumor free throughout the study and survived significantly longer than mice in the other three groups (Fig. 2F). The total flux of individual mice was also plotted and is shown in fig. S8 (A to D). This study was independently repeated, and a total of 10 mice treated with RPE-mIL2 capsules exhibited significant tumor reduction when compared to sham-treated (*n* = 11), RPE-treated (*n* = 11), or rmIL2-injected (*n* = 11) mice. In addition, in an independent ID8-Fluc study, necropsy imaging at day 75 after treatment revealed that all RPE-mIL2–treated mice were healthy and tumor free (fig. S9). Together, these results highlight clear antitumor effects of sustained local delivery of RPE-mIL2 for ovarian cancer.

Before implantation, capsules containing control, naïve RPE cells were assayed via ELISA under the same conditions as the RPE-mIL2 capsules, and no detectable concentration of mIL2 was found in the supernatant. The rest of the biomaterial delivery system was identical to the RPE-mIL2 capsules, allowing us to conclude that therapeutic effects were due to cell-produced mIL2 alone.

To assess the difference in bioactivity between rmIL2 and cell-produced mIL2, we isolated murine T cells and stimulated them with either RPE-mIL2 or rmIL2 for 24 hours. RPE-mIL2 was found to promote T cell viability (intracellular adenosine triphosphate) and proliferation (*27*) 36× more than rmIL2 at low (1 ng/ml) and 128× more at high (10 ng/ml) concentrations (Fig. 2G), demonstrating that RPE-mIL2 was significantly more bioactive than rmIL2. Viability and proliferation were also quantified at 72 hours, and as seen in fig. S10, the isolated T cells were still viable and proliferating in a dose-dependent manner. These data suggest that the cell-produced mIL2 is more potent than its recombinant counterpart. Furthermore, RPE-hIL2 was also found to promote greater human T cell viability and proliferation than either the mouse or human recombinant protein across multiple doses (fig. S11). We hypothesize that this difference in biological activity is likely due to differences in gene sequences and/or glycosylation patterns between the molecules.

### RPE-mIL2 induces cytotoxic T cell proliferation in ovarian cancer mice

**Fig. 3.**
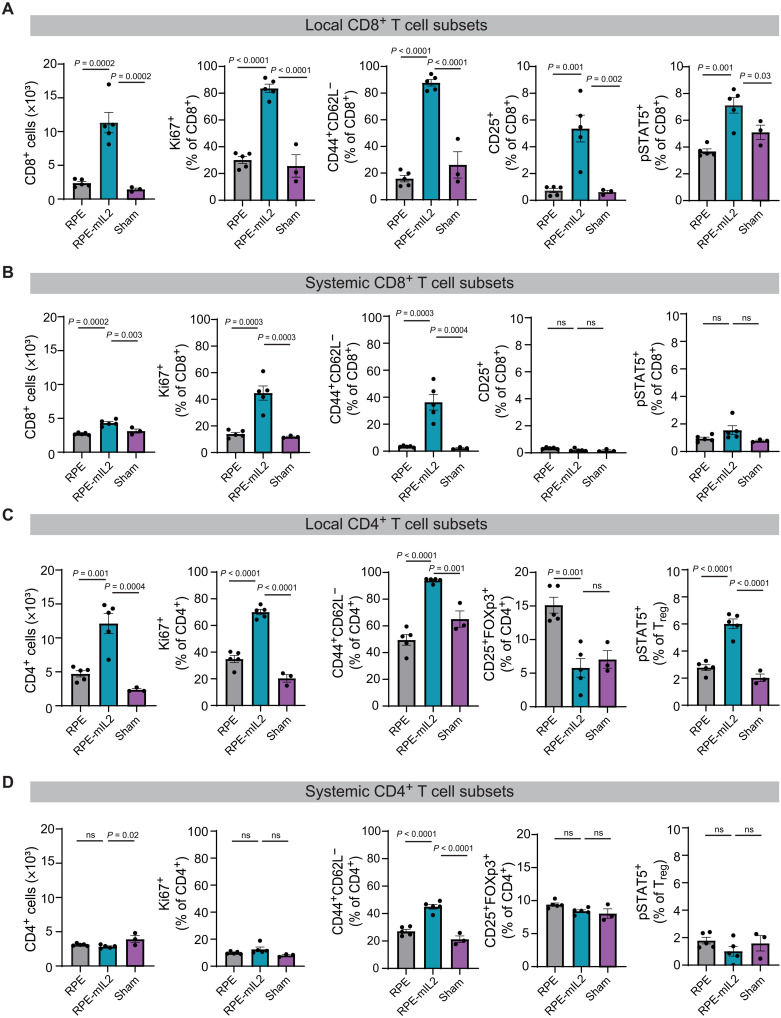
Insights into immunological effects of RPE-mIL2 on tumor reduction in vivo. At 7 days after treatment, mice in treatment groups (RPE versus RPE-mIL2; *n* = 5) or control group (sham; *n* = 3) were euthanized, and the cells of the ascites/IP fluid (local) and spleen (systemic) were stained and analyzed using flow cytometry and then plotted to show the (**A**) local CD8^+^ T cell subsets including CD8^+^ counts or percentage of Ki67^+^, CD25^+^, pSTAT5^+^, or CD44^+^CD62L^−^ T cells as frequency of CD8^+^ T cells; (**B**) systemic CD8^+^ T cell subsets including CD8^+^ counts, percentage of Ki67^+^, CD25^+^, pSTAT5^+^, or CD44^+^CD62L^−^ T cells as frequency of CD8^+^ T cells; (**C**) local CD4^+^ T cell subsets including CD4^+^ counts, percentage of Ki67^+^, CD25^+^, CD44^+^CD62L^−^ T cells, T_regs_ (CD4^+^CD5^+^FOXp3^+^), or pSTAT5^+^ T_regs_ as frequency of CD4^+^ T cells; or (**D**) systemic CD4^+^ T cell subsets including CD4^+^ counts, percentage of Ki67^+^, CD25^+^, CD44^+^CD62L^−^ T cells, T_regs_ (CD4^+^CD5^+^FOXp3^+^), or pSTAT5^+^ T_regs_ as frequency of CD4^+^ T cells. *P* values were determined by ordinary one-way ANOVA with the Holm-Sidak method for multiple comparisons. ns, not significant.

To gain mechanistic insight into the local and systemic immunological changes seen with cell-produced mIL2 from our cytokine factories, we used flow cytometry to survey the immune populations that were activated or proliferating 1 week after treatment. Because high concentrations of IL2 stimulate effector T cells (*42*), we hypothesized that tumor-adjacent delivery of our RPE-mIL2 capsules caused increases in effector T cell (CD8^+^) activation, proliferation, and migration into the tumor space without stimulating regulatory T cells (T_regs_) (CD4^+^CD25^+^FOXp3^+^). To address this question, we studied the immunological landscape of the local (IP space) and systemic (spleen) compartments of ovarian cancer mice using flow cytometry. Our data confirmed that there were at least 4.5× more CD8^+^ T cells in the IP space (local) in the mice treated with RPE-mIL2. Furthermore, we found an increase of at least 2.5× in the activation (CD25^+^CD8^+^) and proliferation (Ki67^+^CD8^+^) of local cytotoxic (CD8^+^) T cells as well as a more than 3× increase in the percentage of CD8^+^ effector (CD8^+^CD44^+^CD62L^−^) cells relative to the control groups (Fig. 3A). Last, we also found a significantly higher percentage of CD8^+^ cells that were able to bind IL2 phosphorylated signal transducer and activator of transcription 5 (pSTAT5^+^) in the IP space (Fig. 3A). Even after only 7 days of treatment, we found at least 1.5× more CD8^+^ T cells in the spleen (systemic) of the RPE-mIL2–treated mice than the control groups (Fig. 3B). Of these systemic CD8^+^ T cells, 45% were proliferating cells (3× more than the control groups) and 36% were effector cells (10× more than the control groups) (Fig. 3B). We did not observe significant T cell activation or pSTAT5 signaling in the spleen at this time point. Together, these data suggest that RPE-mIL2 treatment locally and systemically induces rapid expansion and proliferation of CD8^+^ T cells.

To further investigate the local and systemic immunological landscape, we also evaluated CD4^+^ T cell subsets. We found at least 2.7× more CD4^+^ T cells in the IP space of the RPE-mIL2–treated mice relative to the controls (Fig. 3C). We also found that RPE-mIL2 treatment caused an increase of at least two times in the proliferation (Ki67^+^CD4^+^) of local CD4^+^ T cells. A total of 94% of the CD4^+^ T cells in the IP space of RPE-mIL2–treated mice were effector cells (CD4^+^CD44^+^CD62L^−^), which suggests cytotoxic capacity. Of clinical importance, RPE-mIL2 did not cause substantial expansion of T_regs_, which are known to dampen or counterbalance the cytotoxic capacity of immune cells. However, the T_regs_ that were present locally were still able to interact with IL2, as shown by the pSTAT5 response seen in Fig. 3C. While the RPE-mIL2 treatment did not increase the relative fraction of T_regs_, the existing T_regs_ were indeed responsive to the treatment, as evidenced by the increase in pSTAT5. Thus, any increases in the total number of T_regs_ did not inhibit antitumor immunity, likely because of the concomitant increase in effector T cells. Nonetheless, the maintenance of T_reg_ function should be beneficial in avoiding unwanted inflammation or autoimmunity during resolution of the immune response following tumor clearance. From a systemic standpoint, at day 7 after treatment, we did not see significant expansion or proliferation of CD4^+^ T cells, T_regs_, or pSTAT5 responsiveness in the RPE-mIL2–treated mice (Fig. 3D). This suggests that IL2-based systemic T cell expansion and proliferation preferentially binds to CD8^+^ T cells by day 7. Together, these data suggest that IP administration of RPE-mIL2 locally causes significant activation, expansion, and proliferation of cytotoxic immune cells and systemically induces expansion and proliferation of CD8^+^ T cells in mice with ovarian cancer.

Last, we evaluated the presence and proliferation status of B and natural killer (NK) cells in the local and systemic compartments. We found that the mice in the RPE and sham treatment groups had at least six times more CD19^+^ B cells present in the IP space than the RPE-mIL2–treated mice (fig. S12A). However, of the local population of CD19^+^ B cells in the RPE-mIL2–treated mice, more than 37% were proliferating (fig. S12A). Similarly, we found that less than 4% of local lymphocytes in mice from any group were NK cells, but 88% of the local NK cells in the RPE-mIL2–treated mice were proliferating (fig. S12A). These data suggest that the mIL2 did not cause B or NK cell infiltration but did play a role in inducing B and NK cell proliferation in the IP space. Systemically, we found similar percentages of B and NK cells in the RPE-mIL2–treated mice as seen in the IP space but lower proliferation (21 and 25%, respectively) suggesting that, at this time point, RPE-mIL2 primarily induced B and NK cell proliferation locally in mice with ovarian cancer (fig. S12B). Representative gating strategy for immune cell populations in Fig. 3 can be seen in fig. S13.

### CD8^+^ cytotoxic T cells are essential for antitumor responses seen after RPE-mIL2 treatment in ovarian cancer mice

**Fig. 4.**
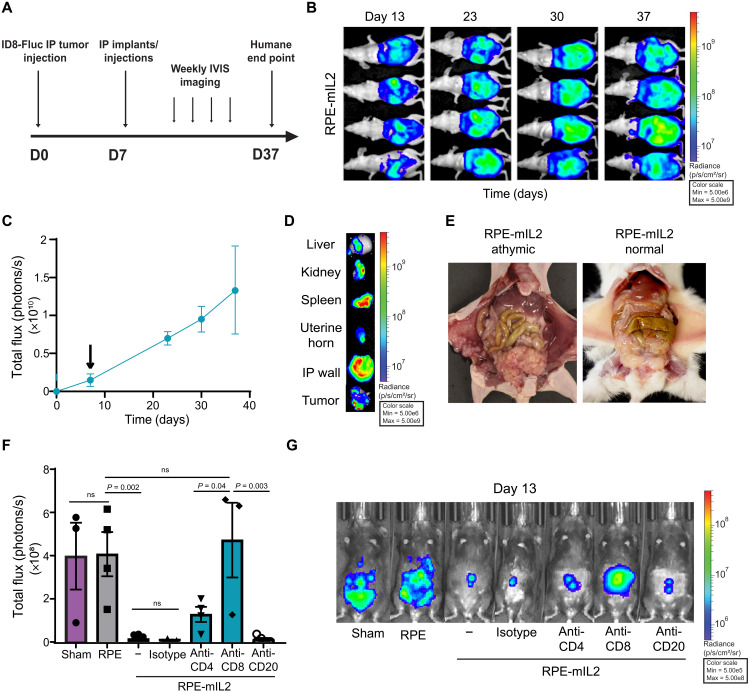
Evaluation of the mechanistic effects of immune populations subsequent to RPE-mIL2 treatment on mice with IP ID8 tumors. (**A**) Experimental timeline for Nu/Nu nude mouse survival study. D0, day 0. (**B**) Luminescent images tracking tumor burden over time. (**C**) Total flux (photons/s) quantified from luminescent images acquired over time and plotted as means ± SEM. The black arrow indicates capsule administration at 7 days after injection. (**D**) Representative ex vivo organ in vivo imaging system (IVIS) images at terminal end points. (**E**) Athymic female mice (*n* = 4) injected with ID8-Fluc and implanted with RPE-mIL2 capsules were photographed at their humane end point and compared to healthy mice treated with RPE-mIL2 (B6 albino; *n* = 5). Photo credit: Maria Ruocco, Rice University (left), and Andrea Hernandez, Rice University (right). No significant deviations from starting body weight were observed following administration of RPE-mIL2 treatment in this study. (**F**) Total flux (photons/s) quantified from luminescent images acquired 6 days after treatment and plotted as means ± SEM. Mice were injected IP with anti-CD4 (*n* = 4) or anti-CD8 (*n* = 3) at days −2, 0, and 2 after treatment. Mice were injected intravenously with anti-CD20 (*n* = 4) at day −2 after treatment. (**G**) Luminescent images of IP tumor burden 6 days after. All data are from (B) to (E), (F), and (G) are from individual dedicated experiments.

Given that the bioactivity and flow cytometry data suggested that our antitumor efficacy results were likely due to activation of cytotoxic T cells from our cell-produced mIL2, as well as B cell proliferation from the implantation of the biomaterial system, we hypothesized that Nu/Nu athymic mice (which lack fully functional T and B cells) with ovarian cancer would not benefit from RPE-mIL2 treatment. Following the experimental conditions in Fig. 4A, we found that ovarian cancer–bearing Nu/Nu nude mice treated with RPE-mIL2 did not exhibit tumor regression. Our data indicated that high-dose RPE-mIL2 treatment did not prevent or even delay tumor growth with the absence of functional B and T cells (Fig. 4, B and C). Ex vivo organ imaging showed extensive tumor burden on all IP organs collected as well as severe tumor masses in the IP space and along the abdominal wall (Fig. 4D). Hemoperitoneum, organ discoloration, and extensive tumor burden were seen during the necropsies (shown next to an albino mouse treated with 200 RPE-mIL2 capsules) (Fig. 4E).

To elucidate which specific immune cell population was responsible for our antitumor efficacy results, we used antibodies against CD4^+^ T cells, CD8^+^ T cells, or CD20^+^ B cells in ovarian cancer–bearing mice treated with RPE-mIL2. As shown in Fig. 4F, mice that were depleted of CD8^+^ T cells were unable to mount a sufficient antitumor response. The average total flux from this group was comparable to mice in the sham and RPE control groups. CD20-depleted mice showed extensive response to RPE-mIL2 treatment, which was similar to the immune-competent mice and the isotype control, suggesting that CD20^+^ B cells are not required for an antitumor response with our treatment (Fig. 4, F and G). However, the CD4^+^ T cell–depleted mice showed a partial response that suggests a secondary role of CD4^+^ T cells in the overall antitumor response to RPE-mIL2 in ovarian cancer mice (Fig. 4, F and G). Together, these data provide mechanistic insight into the immune cells responsible for antitumor efficacy after RPE-mIL2 treatment and suggest that our results are largely CD8^+^ T cell dependent.

### RPE-IL2 eradicates tumor burden in a mouse model of aggressive colorectal cancer

**Fig. 5.**
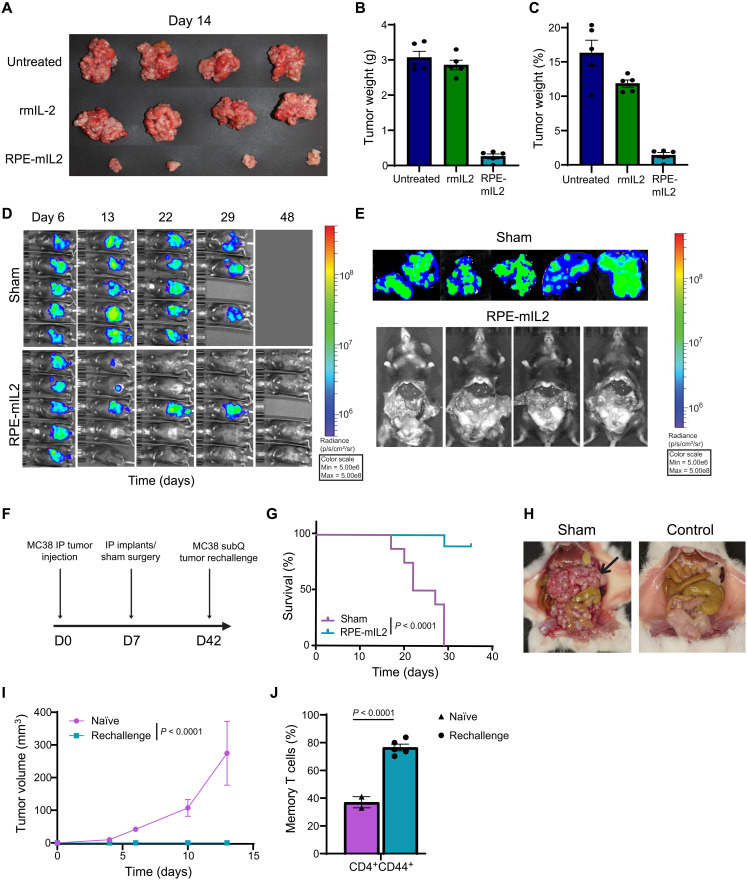
Cytokine secretion from RPE-mIL2 provides tumor reductive effects in an MC38 colorectal cancer model and protection from recurrence. (**A**) Visible IP tumors imaged at 7 days after treatment. Photo credit: Maria Ruocco, Rice University. (**B**) Tumor weights (means ± SEM). *P* values (*P* < 0.001 between untreated versus RPE-mIL2 and rmIL2 versus RPE-mIL2) determined by multiple comparisons test. Here, groups were randomly stratified before implantation. To account for heterogeneous variances, a Brown-Forsythe and Welch ANOVA test was used, with multiple comparisons using Dunnett’s T3 test. (**C**) Tumor weights as a percentage of total body weight (means ± SEM). *P* values (*P* < 0.001 between untreated versus RPE-mIL2 and rmIL2 versus RPE-mIL2) calculated by one-way ANOVA, with Holm-Sidak’s multiple comparisons test. (A) to (C) are representative of two individual experiments. (**D**) Luminescent images (*n* = 5) over time. (**E**) Ex vivo IVIS images of sham tumors (top) and exposed IP cavity of RPE-mIL2 mice (bottom) at day 57 after treatment. (D and E) From two independent experiments. (**F**) Schematic for subcutaneous (subQ) rechallenge study. (**G**) Percent survival over time. *P* value was determined by a comparison of survival curves by the log-rank (Mantel-Cox) test. (**H**) Necropsy imaging of sham MC38 mouse euthanized at humane end point versus naïve mouse. Photo credit: Michael Doerfert, Rice University. Black arrow indicates large tumor mass. (**I**) Subcutaneous tumor volume tracked over time. *P* value was calculated using one-way ANOVA. (**J**) CD3^+^CD4^+^CD44 memory T cell percentages. *P* values were determined by one-way ANOVA. (I) and (J) are from one dedicated experiment.

To test the efficacy of this treatment modality in a mouse model of colorectal cancer, we treated MC38 tumor–bearing mice with RPE-mIL2. MC38 tumors exhibit a high mutational load, are highly immunogenic, and are amenable to many immunotherapy approaches. After only 1 week of treatment, the tissue collected from the RPE-mIL2 did not appear similar in color nor vascularity to the other groups and weighed more than 10× less than the other tumors (Fig. 5, A and B). While the average body weight of the mice in each of the three groups was similar, there was a significant difference in the weight of the tumors extracted from the mice (Fig. 5C). No significant loss of body weight was observed in RPE-mIL2–treated groups, suggesting that the treatment was well tolerated.

The data above suggest that RPE-mIL2 could also be used to elicit tumor reduction in mice with colorectal cancer. To confirm this hypothesis, we used in vivo imaging system (IVIS) imaging to track tumor burden over time. Similar to our results with the ID8-Fluc model, all five of the RPE-mIL2–treated animals showed a significant reduction in tumor burden after only 6 days of treatment, suggesting that RPE-mIL2 rapidly activates the adaptive immune response after implantation. Four of five mice were tumor free by day 15 after injection (Fig. 5D) and remained tumor free for all 57 days of the experiment. As animals in the sham group reached humane end points, visible tumor masses were explanted and imaged ex vivo. As shown in Fig. 5E (top), all five sham mice contained large, invasive tumors in the IP cavity at the time of euthanasia. To further highlight the absence of tumor in the IP cavity of the RPE-mIL2–treated mice, we immediately IVIS-imaged the mice after euthanasia with the IP cavity and all IP organs exposed Fig. 5E (bottom). These images demonstrate the lack of IP tumor growth in the treatment group.

While these results confirmed that RPE-mIL2 has utility for tumor reduction in a second tumor type, they also raise the question regarding whether this system would elicit systemic immunity and protection against tumor recurrence in the treated mice. To test this, we designed a tumor rechallenge experiment (Fig. 5F) where mice with IP tumors previously treated with RPE-mIL2 were challenged with a second MC38 tumor injection at a distant location. The survival study was terminated 8 days after the last sham mouse reached a humane end point (Fig. 5G). A representative image of the severe tumor mass within the IP cavity of sham-treated mice at a humane end point is shown in comparison to a healthy, untreated mouse in Fig. 5H. On day 42, all the treated RPE-mIL2 mice were rechallenged subcutaneously with 5 × 10^5^ MC38 cells and monitored until control mice reached a critical tumor volume (15 mm in diameter). After 2 weeks, zero of five RPE-mIL2 mice developed a subcutaneous tumor, while five of eight naïve mice developed large visible tumors (Fig. 5I). These results suggested that, in addition to increasing activation and proliferation of CD8^+^ T cells, RPE-mIL2 treatment also aids in the development of memory T cells that prevent secondary tumors from developing. This hypothesis was confirmed using flow cytometry. The percentage of CD4^+^ memory T cells (CD3^+^CD4^+^CD44^+^) in the spleen of the RPE-mIL2–treated mice was more than two times greater than that of the control group (Fig. 5J). No significant deviations from starting body weight were observed following administration of RPE-mIL2 treatment in this study. Representative gating strategy for immune cell populations in Fig. 5 can be seen in fig. S14. Together, these results demonstrate clear antitumor effects of continuous local delivery of native mIL2 for colorectal cancer and highlight the ability to induce memory T cell formation to protect against tumor recurrence.

### A simple PK model of RPE-IL2 production and transport explains mouse data and predicts dynamics in human and nonhuman primates

**Fig. 6.**
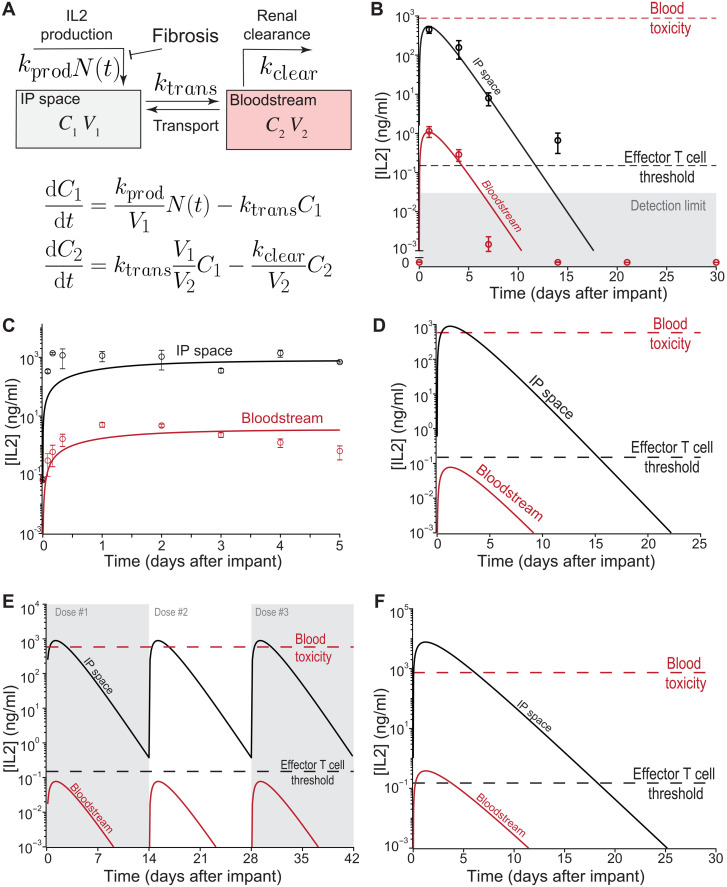
PK model fits mouse data and predicts the IL2 dynamics for human and nonhuman primate administration. (**A**) Schematic overview and governing equations of the two-compartment IL2 PK model. (**B**) Data from in vivo mouse experiments (Fig. 1J) can be well fitted by the model (solid curves). (**C**) The two-compartment model can also be fitted to the IP IL2 infusion clinical trial data from Edwards *et al.* (*34*). The data shown correspond to a dosage of 0.33 mg/m^2^ peritoneal surface area per day; thus, with a typical surface area of ~1.75 (*65*), we assumed a constant IL2 influx of ~0.57 mg/day. The fit results in the estimate human compartment transport rate (*k*_trans_ ≈ 0.7/day). (**D**) Predicted in vivo concentration time course after capsule dosing (*N*_0_ = 5000) using human model parameters. The model predicts that therapeutic levels of IL2 can be maintained for ~13 days. (**E**) Predicted in vivo concentration time course with periodic capsule readministration (*N*_0_ = 5000; 14 days between dosing events) using human model parameters. The parameter values used are the same as in (D). (**F**) Predicted in vivo concentration time course with model extrapolated to apply for NHPs. The estimated human compartment transport rate (*k*_trans_ ~ 0.7/day) was used with NHP-specific model parameters to predict NHP concentration time courses for model validation. Parameter values used in each simulation are shown in table S6.

To better identify PK determinants of dosing of localized IL2 production by RPE-IL2 cytokine factories, we developed a simple linear PK model (Fig. 6A) based on prior work in the literature (*32*). The model includes two compartments for which IL2 measurements are available: the IP space and the systemic circulation (blood). The fluid volumes of each compartment are readily available from anatomic data and injection protocol. The model accounts for the production of IL2 from implanted capsules in IP space, transport between IP space and blood, and renal clearance of IL2 from the blood. To model PFO-induced cell death, we assumed an exponential decrease in the number of producing cells, i.e., *N*(*t*) = *N*_0_^(−λ*t*)^. As a result, the model depends on just four parameters: (i) the IL2 production rate, (ii) the intercompartment transport rate, (iii) the renal clearance rate, and (iv) the capsule decay rate.

As a first step, we constrained the model parameters to physiologically relevant concentrations. Cytokine production rates per capsule were estimated using in vitro IL2 data from ELISA measurements (Fig. 1C). We estimate that the production rate was in the range ~5000 to ~10,000 pg/day per capsule and ultimately used a range of ~5 × 10^3^ to 2 × 10^4^ to constrain the production rate during fitting. The mouse renal clearance was estimated from experimental measurements in rats (*33*), and as a result, a range of ~250 to ~750 ml/min was used for fitting. Explanted capsules (Fig. 1J) began displaying PFO as earlier as 4 days after implantation, which suggested relatively fast capsule decay dynamics. We nevertheless used a relatively wide range of 10^−2^ to 10 day^−1^ to constrain the capsule decay rate, expecting that the correct decay value will be constrained by the fitting procedure.

Our modeling of in vivo data (Fig. 6B) indicated that the half-life of the encapsulated cells in the IP space is only of the order of 1 day. However, the localized production of IL2 during that period is sufficient to keep the IL2 concentration in the IP space above the effector T cell threshold for more than 12 days due to the slow transport of IL2 between the IP space and blood compartments. The gradient of IL2 concentration between the blood and the IP space that allows the system to avoid systemic side effects is due to a two– to three–order of magnitude difference between the time scales of IP-to-blood transport and renal clearance.

To assess the feasibility of clinical translation of the platform, we decided to simulate our model with human parameters. First, we used the modified version of our model (with constant, time-independent infusion rate) to clinical data from Edwards *et al.* (*45*). As a result, we were able to estimate the intercompartment transport rate in humans (Fig. 6C). To our surprise, the time scale of the transport in humans was about the same as in mice, indicating that this rate is unlikely to scale with organ size. However, our modeling also suggests that the large amount of fluid injected into IP space in these experiments led to a decrease in the gradient of IL2 between IP space and blood relative to our mouse experiments. We therefore further evaluated IL2 dynamics following IP implantation of 5000 capsules in 50 ml of carrier fluid (dosage chosen to approximately match the peak IL2 in mouse experiments), using the estimated transport rate, human blood volume from Feldschuh *et al.* (*43*), renal clearance rate from Konrad *et al.* (*44*), and the per-capsule production rate and decay rate from mice data fit. The full table of model parameters is provided in table S6. Predicted human in vivo IL2 dynamics are shown in Fig. 6D. The large IL2 gradient between the bloodstream and the IP space is predicted to provide therapeutic levels of IL2 within the IP space for approximately 13 days while maintaining the IL2 concentration in the circulation sufficiently below systemic toxicity levels to avoid toxic side effects. We additionally simulated a treatment regime involving periodic capsule readministration to maintain IL2 concentrations above the effector T cell threshold (Fig. 5E); the model predicts that, with three dosing cycles every 14 days, the IL2 concentration within the IP space can be maintained above therapeutic levels for more than 40 days. The dosing regimen can be optimized using the PK model, for example, optimizing the dosing period for a given capsule dosage to maintain IP IL2 concentrations above a target concentration threshold (fig. S15A). Given the linear scaling of the peak IP IL2 concentration predicted by the model (fig. S15B), the treatment regimen can be designed both to maintain therapeutic levels of IL2 and to avoid excessively high IL2 levels.

To further challenge the predictive capacity of the model, we simulated the results of cytokine factory implantation into nonhuman primates. Given the similarity of the intercompartment transport rate in mice and humans, we fixed the NHP transport rate to be equal to the transport rate estimated from the data of Edwards *et al.* (*34*). The per-capsule IL2 production rate and the capsule decay rate were estimated from fits to the mouse data, and the NHP renal clearance rate was estimated Zhang *et al.* (*45*). The NHP blood volume was set on the basis of available anatomical data (*46*), and the IP fluid volume was set on the basis of the IP fluid:blood ratio in humans. With these parameter estimates, we predicted what would occur in an NHP (Fig. 6F). The model prediction suggests that therapeutic concentrations of IL2 can be maintained for around 13 days in NHPs with an initial dose of 1795 capsules (~4 ml of volume).

### RPE-hIL2 produces an IL2 PK/PD profile predictive of human efficacy and is well tolerated in nonhuman primates

A key design consideration for the RPE-hIL2 cytokine factories is their clinical translatability. IP delivery of anticancer agents is a cornerstone in patients with peritoneal metastatic disease, and hence, there are numerous clinically available devices to facilitate IP delivery. To further mimic clinical IL2 dosing and ensure accurate dosing, we transitioned from dosing by capsule number to dosing by IL2 production from each capsule. To do this, we multiplied the number of capsules administered by the IL2 production from an individual capsule over 24 hours. To evaluate the clinical translation of our product, we used cynomolgus macaques because they are bipedal and more immunologically similar to humans than rodents and thus are a more appropriate surrogate model to study before translating to human patients (*47*, *48*). Similarly, the cynomolgus macaques’ FBR to immunostimulatory implants more closely resembles the response seen in humans because of their exposure to a more diverse set of antigens than inbred rodents (*47*).

**Fig. 7.**
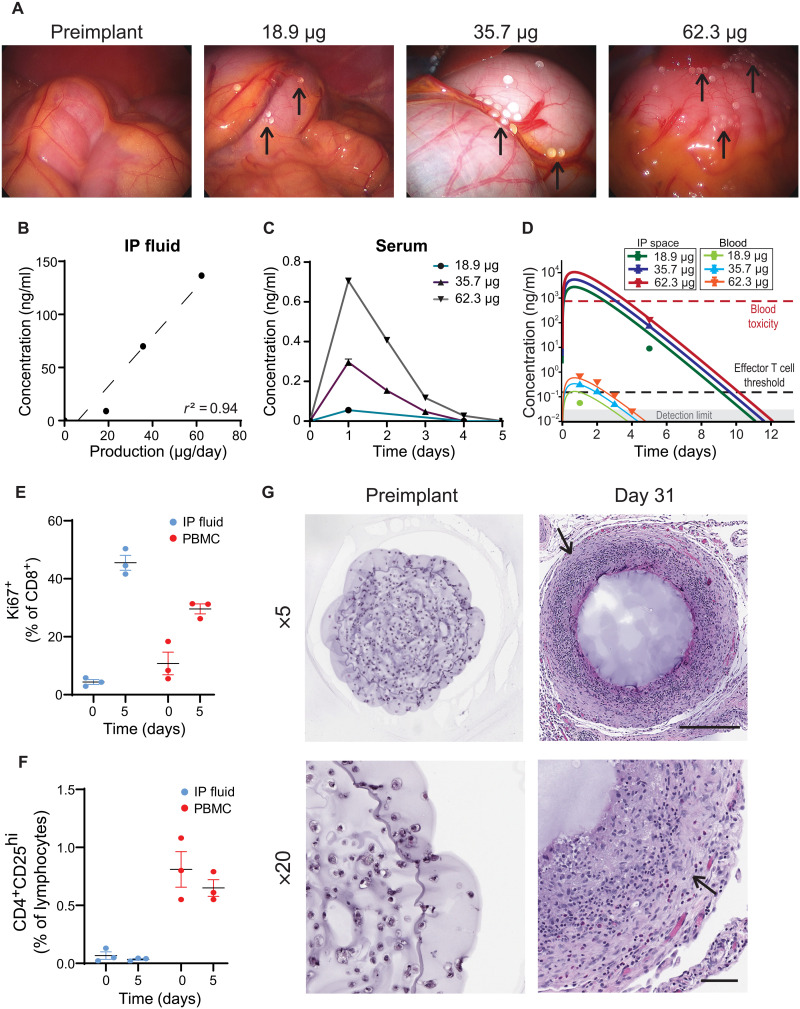
RPE-hIL2 dosing is predictable in nonhuman primates with immune system stimulation comparable to responses seen in rodents. (**A**) Laparoscopic images of the IP space of nonhuman primates before implant and after implantation of each of three doses (*n* = 1 per dose). Capsules can be seen on and around IP organs (black arrows). Photo credit: Peter Rios, CellTrans Inc. (**B**) hIL2 concentration in IP fluid of each primate at day 5 after implant is linear with *r*^2^ = 0.94, calculated in GraphPad Prism. (**C**) hIL2 concentrations (ng/ml) in blood over time. (**D**) Model simulation using parameters obtained from fitting to the NHP data in (B) and (C). Changes in (**E**) are the percentage of CD8^+^Ki67^+^ cytotoxic T cells as frequency of CD8^+^ cells and (**F**) the percentage of CD4^+^CD25^hi^ T_regs_ as frequency of lymphocytes in the IP fluid and blood from days 0 to 5 after treatment (*n* = 3). PBMC, peripheral blood mononuclear cell. (**G**) Hematoxylin and eosin (H&E) staining of explanted capsules at various time points. Capsule sections are shown at ×5 magnification and ×20 magnification. Scale bars, 500 μm (×5) and 100 μm (×20). Host immune cells accumulate on the surface of the capsules over time (black arrows). All data are from one dedicated experiment.

In the first study, we administered a low (18.9 μg hIL2/day), medium (35.7 μg hIL2/day), and high (62.3 μg hIL2/day) dose of RPE-hIL2 capsules into the IP space of nonhuman primates and assayed for local and systemic hIL2 concentrations to validate our ability to control dosing in a large animal model. The volume taken up by each of the doses of capsules (black arrows) inside the primate is a small fraction of the available space in the IP cavity (Fig. 7A). On day 5, the IP fluid hIL2 concentration varied linearly (*r*^2^ = 0.94) with the dose of hIL2 administered (Fig. 7B), and the peak blood hIL2 concentrations (day 1) were at least 160× lower than the IP fluid concentration, suggesting the feasibility of controlling the local and systemic cytokine concentration over time with our system (Fig. 7C).

To better understand the dynamics of IL2 in the blood and IP space over the implantation period, we used the data from Fig. 7 (B and C) to estimate the PK model parameters for the NHP experiments. The results demonstrate excellent agreement with the highest two doses and allow us to predict the peak levels in the blood and IP space for each animal. Overall, the fitted NHP time courses (Fig. 7D) were qualitatively very similar to our initial predictions (Fig. 6F). The predicted and fitted time courses both displayed a very similar peak value and comparable IP:blood ratio (~10,000-fold difference between the IP and blood IL2 concentrations). However, the time scale of the dynamics was slightly different: The predicted therapeutic window was approximately 18 days for the highest dose (1795 capsules), whereas the projected IP time course from the fit suggested a therapeutic window of only 11 days. This difference is largely due to the difference in kinetic parameters in each simulation; the transport rate (*k*_trans_) and capsule decay (λ) from the NHP fit were approximately 50% greater than the parameters used in our initial prediction and subsequently resulted in a faster time scale of IL2 elimination. Nevertheless, the results demonstrate that both transport rate changes very little (less than a factor of 2) between mouse data fit, human estimation from clinical injection studies (*34*), and NHP experiments fit.

Our dose-finding study led us to hypothesize that, similar to the mouse studies, RPE-hIL2–related induction of cytotoxic T cell proliferation should be accompanied by little to no expansion of T_regs_. Three additional primates received RPE-hIL2 (35.7 μg hIL2/day) surgically administered in the IP space. Five days after capsule implantation, IP cells and peripheral blood mononuclear cells were isolated and analyzed using flow cytometry. More than 40% of the CD8^+^ T cells in the IP fluid and 30% of the systemic CD8^+^ T cells were proliferating (Fig. 7E), further highlighting the ability of the RPE-hIL2 to activate T cells in the nonhuman primate. In addition, the hIL2 dose was still high enough to prevent T_reg_ expansion within the IP cavity or in the systemic circulation (Fig. 7F). Representative gating strategy for immune cell populations in Fig. 7 can be seen in fig. S16. These data suggest our systems’ ability to preferentially induce immune cell activation at a tolerable dose that is of clinical importance for a successful cancer therapy. By day 31, an extensive cell coating was seen on the surface of capsules, and the cells inside the capsules could no longer be detected (Fig. 7E).

**Fig. 8.**
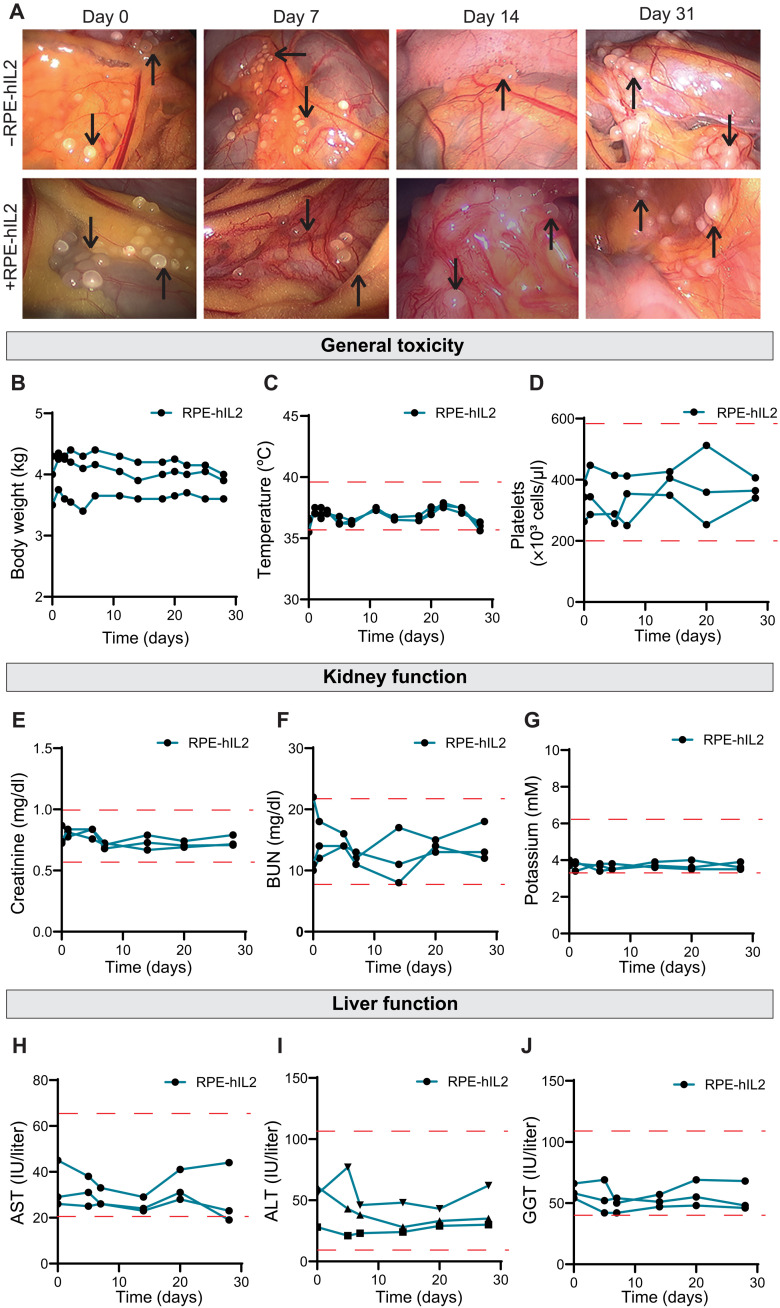
RPE-hIL2 is well tolerated in nonhuman primates. (**A**) Laparoscopic images of the IP space of nonhuman primates given either +RPE-hIL2 capsules (*n* = 1) or capsules containing naïve RPE cells (−RPE-hIL2; *n* = 1) over time. Capsules can be seen on and around IP organs (black arrows) and become fibrosed over time. Photo credit: Peter Rios, CellTrans Inc. Evaluation of general toxicity through changes in (**B**) body weight, (**C**) temperature, and (**D**) number of platelets over time (*n* = 3). Evaluation of kidney function through changes in (**E**) creatinine levels, (**F**) BUN levels, and (**G**) potassium levels over time (*n* = 3). Evaluation of liver function through changes in (**H**) AST levels, (**I**) ALT levels, and (**J**) GGT levels over time (*n* = 3). Dashed lines represent healthy range for the respective measurements. All data are from one dedicated experiment.

Upon alginate implantation, there is transient immune activation that resolves over time, leading to stable PFO around capsules. To study how the FBR seen in the mouse studies translated into the cynomolgus macaques and validate that the immune response was not specific to the RPE-hIL2 secretion, we dosed two additional primates with cytokine factories. The extent of PFO on the surface of the capsules was monitored using hematoxylin and eosin (H&E) staining. One primate was given capsules containing naïve RPE cells, and the other was given RPE-hIL2 capsules. On days 0, 7, 14, and 31, laparoscopic imaging was used to visualize the capsules inside the IP cavity of each primate (Fig. 8A). Both primates exhibited PFO on the surface of the capsules by day 14, as seen by the opaque white coating (Fig. 8A), suggesting that the FBR translated directly into primates.

Last, one of the biggest clinical limitations of IL2 is the toxicity seen in clinical trials. To assess the safety and toxicity profile of our implantable cytokine factories, we conducted two RPE-hIL2 administration studies in cynomolgus macaques. The primary organs implicated in IL2-related toxicities include the liver, kidney, and lungs (*49*), and for this reason, we used H&E staining to assess the histopathologic condition of these organs for the highest hIL2 dose administered (fig. S17). The liver was noted as showing centrilobular hepatocytes slightly vacuolated but was not of clinical concern. In addition, lung and kidney histology did not exhibit notable changes, suggesting that RPE-hIL2 capsules were well tolerated by the primate. Furthermore, complete blood count and blood chemistry analysis at days 7 and 28 show no significant deviations from healthy ranges for red blood cell, white blood cell, lymphocyte, and neutrophils counts nor significant deviations in temperature, weight, blood urea nitrogen (BUN), creatinine, and potassium levels, further suggesting that all doses were well tolerated by the primate (tables S7 to S12). Healthy reference ranges for complete blood counts were generated using the ADVIA 120 hematology system (Siemens). Healthy reference ranges for blood chemistries were generated internally at the University of Illinois-Chicago (UIC) Biologic Resources Laboratory from a sample of 19 healthy cynomolgus monkeys using the AU480 chemistry analyzer (Beckman Coulter). Healthy ranges for creatinine levels were taken from the study of Xie *et al.* (*53*).

To further assess whether our platform caused toxicity in nonhuman primates, we evaluated body weight, temperature, platelet count, and kidney and liver function using complete blood count and blood chemistry analysis for 28 days in three primates that were dosed with the medium RPE-hIL2 dose (35.7 μg hIL2/day). We found no significant changes in body weight (Fig. 8B) or deviations outside of the healthy ranges for body temperature (Fig. 8C) or platelet count (Fig. 8D). To assess kidney function, we evaluated potassium, creatinine, and BUN levels over time and similarly found no significant deviations from healthy ranges (Fig. 8, E to G). Last, to assess liver function, we evaluated aspartate aminotransferase (AST), alanine aminotransferase (ALT), and gamma-glutamyl transferase (GGT) levels for 28 days. Again, we found no significant deviations from healthy ranges (Fig. 8, H to J), suggesting that RPE-hIL2 was well tolerated by all primates. In total, seven of seven primates dosed with RPE-hIL2 managed the IP cytokine factory without complication. Together, these data suggest that RPE-hIL2 can stimulate cytotoxic T cell expansion and proliferation without inducing T_reg_ expansion or signs of toxicity in nonhuman primates.

## DISCUSSION

Recent advancements in immunotherapy have been postured to substantially improve cancer survival rates for many forms of cancer (*51*). However, broad clinical use of these approaches still requires innovations to deliver immunotherapy agents with tight spatial and temporal control over the local tumor microenvironment (*52*). Many proinflammatory cytokines including IL2, IL7, and IL12 have been tested for antitumor efficacy in human clinical trials, but these injectable formulations cannot fully eradicate tumors without severe off-target effects and thus remain a major challenge (*1*, *5*, *6*). This deficiency has led to an increase in diverse monotherapeutic as well as combination therapy studies including cytokines, checkpoint inhibitors, cytokines with checkpoint inhibitors, STING (stimulator of interferon genes) agonists, CAR (chimeric antigen receptor)–T cells, TLR3 (Toll-like receptor 3) agonists, and more (*51*, *53*). However, as described by Leach *et al.*, each component of a therapy must have the ability to be partially effective individually for a combination therapy to reach full potential (*53*). We addressed these issues by designing cytokine factors, consisting of microencapsulated cells that can be used for local in vivo delivery of mono- as well as combination therapies.

Encapsulated cell therapies are actively being explored in other preclinical studies. Key examples include the promising work from Schukur *et al.*, where implantable synthetic cytokine converter cells were investigated for the treatment of psoriasis (*54*). Nevertheless, this system design is not amenable for rapid translation into the clinic. Here, we present a highly modular, clinically translatable cytokine factory design that does not depend on receiving stimulus from the host and continuously produces de novo, natural cytokines. By carefully considering the necessary design criteria needed for use in clinical oncology, our system stands apart from others in the immuno-oncology space. It accomplishes a highly desired goal by incorporating a cell engineering technique that simultaneously allows for the integration of a clinically relevant human cell line, prediction of in vivo concentrations in multiple compartments, the ability to accurately dose and redose, an efficient tumor microenvironment delivery paradigm, and a clinically translatable hydrogel formulation. Together, these components help ensure rapid translatability of our platform to human patients with cancer, and this platform was found to be tolerated and efficacious in rodent and nonhuman primates. As seen in the data presented here, both the proinflammatory alginate and the proinflammatory cytokines delivered are needed for the full therapeutic response. Future studies will focus on decoupling the specific role that each component plays in the overall success of the therapy.

Previous studies have shown that alginate can be used to encapsulate various types of cargo, such as cells and soluble drugs, and allow for the delivery of small molecules in vivo (*54*, *55*). In human studies, the natural FBR has been previously shown to cause fibrotic overgrowth on the surface of the capsules that leads to a slow decrease in pore size and eventual cell death when oxygen and nutrients cease to diffuse inside (*38*). Although the alginate does not degrade in vivo, it is biocompatible and has been implanted in mice, primates, and humans for extended time frames without complication. Because the therapeutic cells are confined in dual-layer capsules, the FBR provides a natural and predictable kill switch without the need for administration of small-molecule drugs such as AP1903 for iCasp9 systems or ganciclovir (GCV) for Herpes simplex virus thymidine kinase (HSV-TK) systems (*56*), which often fails to kill 100% of the intended cells. We have rationally designed a robust dosing regimen that benefits from decreases in production over time. In both the rodent and primate studies, we have shown that the cytokine factories become coated with fibrotic overgrowth within 1 month, and the local and systemic cytokine concentrations consistently return to baseline levels, negating the need for an additional kill switch.

Specific advantages of our cell engineering platform include the safe toxicity profile and predictable pharmacokinetics that we observed in our nonhuman primate studies. We show in Figs. 7 and 8 that all three doses administered were well tolerated, provided predictable local concentrations, and resulted in gradual IL2 production shut off due to the FBR to the implanted material. Moreover, our PK model successfully fit the experimental data and provides a mechanistic explanation of the large gradient between blood and IP level of cytokines. The gradient is due to a several–orders of magnitude difference between the time scales of transport into the blood and of renal clearance. Notably and somewhat unexpectedly, the modeling suggests that there is little variation in the transport rate between organisms. This implies that the gradient between IP and blood will be higher in human clinical trials as the clearance rate scales with organism mass.

Clinical success of cytokine immunotherapies require a technology that improves their therapeutic index (*57*). We have developed a unique IL2 delivery platform and carefully compared it to FDA-approved and clinical-stage IL2 delivery platforms in terms of preclinical and clinical efficacy as a monotherapy, potency, local cytokine concentration, half-life, controlled dosing and redosing, duration of activity, and limited toxicity in table S13. Comparative approaches include mRNA-based platforms developed by BioNTech to modify cells in vivo (NCT04455620) (*57*); adenovirus-based platforms designed by Ziopharm (NCT03330197) to deliver recombinant cytokines in vivo, pegylated proteins like NKTR-214 (*12*), and fusion-proteins such as ALKS 4230 (*58*) to increase circulation; selectively activated proteins such as BAY 50-4798 (*59*, *60*); and various antibody platforms such as CEA-IL2v designed by Roche to block IL2 binding to T_regs_ (*59*, *61*). Notably, we have shown that, by providing a continuous supply of naturally produced, bioactive cytokines directly to the local tumor microenvironment from within our hydrogel platform, we could improve the overall therapeutic index of IL2 and allow for readministration of additional doses, if needed. This allows for constant and specific control over the activation states of immune cells directly adjacent to the tumor mass.

In conclusion, here, we present a modular proinflammatory cytokine factory that is safe, predictable, and effective for cancer treatment. Our results are supportive of translation into human clinical trials for ovarian and colorectal cancers. This treatment has the potential to greatly affect the outlook of patients with ovarian cancer with platinum-resistant tumors who currently expect no more than 12 months of median survival with currently available therapies. The system described here enables the rapid development of novel cytokine therapeutics with similar pharmacokinetics without the need for cumbersome protein engineering or extended-release formulation development. The approach developed here can be further generalized to other compartments that exist throughout the body including the pleural space, subcutaneous sites, and tumor resection cavities. These cavities have similarly been explored for localized drug delivery (*62*). For example, continuous intrapleural IL2 infusion has also been evaluated in clinical trials for the treatment of mesothelioma, and intrapleural cavity IL2 levels were measured to be 6000-fold higher than systemic leading to improvements in objective response rate and disease control rate (*63*, *64*). This robust platform can be leveraged to create combination therapies or continue exploring additional monotherapies in cancer types not addressed here, such as cancers of the lung, breast, and brain and other cancers. Alginate-based encapsulation systems for delivery of native IL2 as a successful monotherapy for metastatic cancers is a starting point for encapsulated cell therapies and is essentially only limited by the size of the pores restricting efficient diffusion into the surrounding space. The precise control of IP therapeutic concentrations is a unique feature of our encapsulated cell therapy platform, which is qualitatively not achievable by biologic injections. This technology provides the field with an invaluable tool that can be easily modified and leveraged for other peritoneal cancers including renal, liver, pancreatic, and cervical cancers in future clinical studies and could enable further exploration of therapeutics with substantial toxicity at narrow concentration windows where they are both safe and effective.

## MATERIALS AND METHODS

### Cell culture and engineering

Cell culture media and associated reagents were purchased through Thermo Fisher Scientific. Lipofection reagents (Lipofectamine 3000) and selection media (puromycin) were purchased from Invitrogen. Expression vectors and helper plasmids were developed and purchased through VectorBuilder. CellTiter-Glo 3D (Promega) was used to determine cell counts within capsules. LIVE/DEAD stains (Thermo Fisher Scientific) were used to determine the cell viability of encapsulated cells.

ID8/MOSEC (EMD Millipore, Sigma-Aldrich), human ARPE-19 [American Type Culture Collection (ATCC)], and MC38 (Kerafast) cells were obtained commercially. Human ARPE-19 cells (lot #70022669) have been characterized by ATCC by cytogenetic analysis to be diploid and have the following Short Tandem Repeat (STR) profile: Amelogenin: X,Y; CSF1PO: 11; D13S317: 11,12; D16S539: 9,11; D5S818: 13; D7S820: 9,11; TH01: 6,9.3; TPOX: 9,11; and vWA: 16,19. These cells were cultured using Dulbecco’s modified Eagle’s medium (DMEM/F-12), with 10% fetal bovine serum (FBS) and 1% antibiotic-antimycotic (AA). The medium was changed three times weekly. Medium used for MC38 cells was high-glucose DMEM, 10 mM Hepes, 10% FBS, and 1% AA. Medium used for ID8 cells was DMEM high glucose, 4% FBS, 1% AA, and 1% ITS (Insulin, Transferrin, Selenium).

### Cell transfection/transduction

Plasmids for lentiviral transduction and Lipofectamine transfections of all cells used were designed and purchased from VectorBuilder. ARPE-19 cells were engineered to express cytokines of interest. ID8/MOSEC and MC38 cells were engineered to express firefly luciferase. Engineered cells were assayed for linearity between luciferase expression and cell number using IVIS imaging (methods followed small-animal imaging described below.) ARPE cytokine-expressing cell lines were assayed for cell viability and changes in doubling time after transfection/transduction using traditional cell subculture methods and trypan blue–based counting methods.

### Transfection

For transfection of human and mouse cytokines, ARPE-19 cells that had been passaged four times were seeded into six-well plates at a concentration of 500,000 cells per well. The plate was incubated overnight at 37°C in a 5% CO_2_ humidified atmosphere. Twenty-four hours following seeding, each well was primed with 2 ml of Opti-MEM serum-free medium (Thermo Fisher Scientific). A 2:1 ratio of expression vector to helper plasmid was used to transfect the cells. All transfections were run according to protocols provided by the manufacturer. After incubation at 37°C for 4 hours, the transfection medium was replaced with fresh culture medium containing 10% FBS and 1% AA. After transfection, cells were selected for expression with puromycin for 1 week and then maintained using normal cell culture techniques.

### Transduction

ID8/MOSEC and MC38 cells were transduced using a standard third-generation lentivirus protocol. Lentivirus was produced by transfection of three packaging plasmids (pMLg/PRRE, pRSV-Rev, and pMD2.g) together with an expression vector in human embryonic kidney (HEK) 293T cells. Six to 8 hours after transfection, the cell medium was replaced, and cells were cultured for 36 to 48 hours. The lentivirus was concentrated and used with polybrene to transduce the cells. After a 24-hour incubation with the virus, the medium was changed, and the cells were cultured for 3 days. On the fourth day, cells were selected for expression with puromycin for 1 week and then maintained using normal cell culture techniques.

### Core shell cell encapsulation

Before encapsulation, all buffers were prepared and sterilized through autoclaving and sterile filtering through a vacuum filter. Alginate solutions dissolved at 1.4% (w/v) in 0.8% saline and sterile-filtered through a 0.2-μm syringe filter. The alginate endotoxin levels were less than or equal to 100 EU/g. Cells were trypsinized and collected into a 50-ml conical tube and washed three times with calcium-free Krebs solution. The cells were centrifuged at room temperature at 250*g* for 5 min. After the second wash, the supernatant was aspirated, and cells were resuspended in alginate at a concentration of 10.5 × 10^6^ cells/ml. Encapsulation occurred using a custom-built, two-fluid coaxial electrostatic spraying device. The device consisted of two syringe pumps (Harvard Apparatus), a coaxial nozzle (Rame-Hart), and a voltage generator (Gamma High Voltage) that was attached to the tip of a coaxial needle and grounded to a glass dish containing a 1:4 barium chloride:mannitol cross-linking bath. Alginate droplets were expelled from the coaxial needle into the cross-linking solution at a rate of 5 to 6 ml/hour. The core syringe contained the cells suspended in alginate. The size of the capsules was maintained by adjusting the voltage on the generator; a voltage of ~5.30 kV consistently produced capsules that were 1.5 mm in diameter. Capsules were maintained in the cross-linking bath for 15 min to ensure complete cross-linking. They were subsequently washed three times with Hepes buffer and transferred to a flask containing complete cell culture medium and maintained with normal cell culture techniques.

### Cell viability after encapsulation

Following encapsulation, a subset of capsules were washed with 5 ml of Dulbecco’s phosphate-buffered saline (DPBS) and stained using a stock 2 μM Calcein AM (Calcein acetoxymethyl ester) and 4 μM EthD-1 in DPBS. The sample was incubated for 20 min and imaged using a fluorescence microscope. Twenty capsules were imaged from each sample group of encapsulated cells and examined for live and dead cells; a green fluorescent protein filter was used to capture live cells, and a Texas Red filter was used to capture dead cells.

### Enzyme-linked immunosorbent assay

A total of 10,000 free cells or a single capsule was added to a 96-well plate (*n* = 5 to 8) in 200 μl for 24 hours at 37 degrees in a 5% CO_2_ humidified atmosphere. Cell supernatant was collected from each well and assayed. ELISAs were obtained commercially for mIL2 (BioLegend), hIL2 (R&D Systems), mIL10 (BioLegend), mIL12 (BioLegend), and mIL7 (R&D Systems). The assay was run according to the manufacturer’s protocols. All samples were run in triplicate.

### Cell proliferation assay

RPE and HEK cell aggregates were made using six-well AggreWell400 microwell culture plates (catalog no. 34425). Once spheroids were harvested, they were resuspended in SLG20 alginate (PRONOVA). Core-shell hydrogels were made and subsequently plated into a 96-well plate. Proliferation of the cells within the capsules was measured using a Click-iT EdU (5-ethynyl-2′-deoxyuridine) imaging kit. A 1× working solution of EdU diluted in medium was added to each capsule. At 0, 24, and 72 hours and 7 days, the medium was removed, and capsules were fixed in 4% paraformaldehyde. Capsules were washed with 3% bovine serum albumin (BSA) and permeabilized using 0.5% Triton X-100. To stain the cells, 0.2 ml of the Click-iT reaction cocktail was added to each capsule and incubated for 30 min. The capsules were subsequently washed with 3% BSA. Last, cell nuclei were stained using Hoechst 33343 (NucBlu). Capsules were imaged with an EVOS XL microscope at ×4 magnification.

### T cell viability and proliferation assay

The EasySep T Cell Isolation Kit (STEMCELL Technologies) and associated reagents and devices were used to isolate T cell from C57BL/6 mouse spleens (Jackson Laboratory or Charles River Laboratories). Cells were plated in 96-well plates at 10,000 cells per well in 100 μl of RPMI 1640. Cells were supplemented with cell RPE-mIL2 supernatant (0.1, 1, 5, or 10 ng/ml) quantified via ELISA or recombinant IL2 (1 or 10 ng/ml) (Miltenyi). Twenty-four or 72 hours after incubation at 37°C, cell proliferation (and viability) was measured using the CellTiter-Glo Luminescent Cell Viability Assay (Promega).

### HEK-Blue assay

HEK-Blue IL2 cells and associated reagents were purchased from Invivogen. For assay, 2.5 × 10^4^ HEK-Blue IL2 cells were plated in wells of a 96-well plate in 180 μl. A total of 20 μl of varying concentrations between 10^−3^ and 10^2^ ng/ml of RPE-hIL2 quantified via ELISA or World Health Organization (WHO) standard recombinant hIL2 was added to each well. Cells were incubated for 24 hours and then assayed according to the manufacturer’s instructions.

### Flow cytometry

For analyzing immune cell populations, all anti-mouse and antihuman antibodies are listed in tables S1 to S5. Intracellular staining was accomplished using the FOXp3/Transcription Factor Staining Buffer Set (catalog 00-5523-00, eBioscience) and the BD Cytofix/Cytoperm fixation/permeabilization solution kit (catalog 554714, BD Bioscience).

All antibodies were commercially obtained and prepared the day of staining and maintained in the dark at 4°C or in ice. A total of 10 ml of IP fluid was collected from animals at explant. The 10 ml of IP fluid was filtered through a 70-μm strainer, spun down (2000 rpm, 3 min, room temperature), and then resuspended in 3 ml of ACK (Ammonium-Chloride-Potassium) lysis buffer (catalog A1049201, Thermo Fisher Scientific). Cells were incubated in ACK lysis buffer for 5 min at room temperature. Samples were spun down (500*g*, 5 min, room temperature) and resuspended in 300 μl of fluorescence-activated cell sorting (FACS) buffer. Samples were plated at 100 μl per well per sample. The plates were then spun down (500*g*, 5 min, 4°C), and the supernatant was discarded. A total of 50 μl of 1:100 Fc blocking antibody in FACS buffer was incubated at room temperature for 10 min. A total of 50 μl of surface staining antibody cocktail was added directly to each well on both plates. The plates were incubated at 4°C for 30 min in the dark. A total of 200 μl of FACS buffer was added to wash the samples and then spun down (500*g*, 5 min, 4°C). To the Ki67 antibody panel, 100 μl of BD Cytofix/Cytoperm solution was added, and the samples were incubated at room temperature for 30 min. For the FOXp3 panel, 100 μl of FOXp3 permeabilization solution was added, and the samples were allowed to incubate at room temperature for 60 min. After incubation, 200 μl of BD Cytoperm wash buffer was added to the Ki67 panel, and FOXp3 wash buffer was added to the FOXp3-containing panel. Plates was resuspended in 100 μl of Cytoperm wash buffer and spun down (500*g*, 5 min, 4°C). Pellets were resuspended in 50 μl of their corresponding intracellular staining mixture. Samples were incubated for 30 min at 4°C in the dark. The samples were washed by adding 200 μl of BD Cytoperm wash buffer for Ki67-stained samples and 200 μl of FOXp3 wash buffer for FOXp3-stained samples. Single-color controls and a negative unstained control were prepared for each analysis.

### Animal studies

#### Mouse studies

C57BL/6, C57BL/6 albino, or Nu/Nn nude mice (Jackson Laboratory or Charles River Laboratories) were used for in vivo studies. All animal experiments were approved by Rice University’s Institution Animal Care and Use Committee (IACUC). All biological samples implanted into animals were approved by Rice University’s Institutional Biosafety Committee. For IP tumor models of ID8-Fluc, 5 × 10^6^ or 10 × 10^6^ cells suspended in Hanks’ balanced salt solution (HBSS) were intraperitoneally injected to the lower right abdomen of female mice. To develop IP tumor models of MC38-Fluc, 1 × 10^6^ cells suspended in HBSS were intraperitoneally injected to the lower right abdomen of mixed-gender cohorts of animals. For all IP tumor models, tumors were injected and allowed to develop in vivo for 1 week before treatment (Figs. 2, A to F; 3, A to D; 4, A to G; and 5, A to G). For all studies using IVIS imaging for tumor growth tracking, mice were imaged and stratified into treatment groups 1 day before surgery using the methods described in the “IVIS imaging” section below (Figs. 2, A to F; 3, A to D; 4, A to D; and 5, D to G). Mice were euthanized at humane end points if any of the following criteria were met: (i) weight loss or gain of >20%, (ii) moribund, (iii) severe abdominal swelling, or (iv) jaundice. For rechallenge colorectal studies, 5 × 10^5^ cells suspended in HBSS were injected subcutaneously into the rear flank of C57BL/6 albino male and female mice that showed complete remission from IP tumor inoculations.

For immune cell depletion studies (Fig. 4, F and G), isotype control (LTF-2), anti-CD8a (2.43), or CD4 (GK1.5) antibodies were administered via IP injection at a dose of 100 μg per animals at days −2, 0, and 2 after RPE-mIL2 implantation. These antibodies were purchased from Bio X Cell. For B cell depletion, anti-CD20 (SA271G2) antibodies were administered via intravenous injection at a dose of 250 μg per animal at 2 days before RPE-mIL2 implantation. These antibodies were purchased from BioLegend.

For in vivo kinetic studies (Fig. 1, J and K), 200 RPE-mIL2 capsules (26.2 μg/day) were surgically implanted into the IP space of C57BL/6 mice for 1, 4, 7, 14, 21, or 30 days. Five mice were euthanized at each time point, and IP fluid and blood were assayed for mIL2 levels via ELISA.

#### Experimental controls

rmIL2: All mice injected with recombinant IL2 were injected intraperitoneally at a dose of 250,000 IU/day for three consecutive days. Sham: All mice given sham surgery received the IP surgery but were administered 1 ml of sterile saline. RPE: RPE capsules contained the same density of cells as RPE-mIL2 and RPE-hIL2 capsules but contained unengineered cells. All mice treated with control RPE capsules received IP surgery and were administered 200 RPE capsules.

#### Subcutaneous tumor growth tracking

For subcutaneous MC38 rechallenge models, the tumor size was measured using a digital caliper, and tumor volume was calculated using the formula *V* = 0.5 × (height) × (width^2^).

#### IP tumor growth tracking

Animals injected with ID8-Fluc cells or MC38-Fluc cells were imaged using IVIS 6 days after injection and stratified into experimental groups on the basis of luminescent signal. After surgery, animals were tracked for tumor growth or reduction using IVIS imaging one time per week. Imaging methods are expanded below.

#### IP surgical implantation of capsules in mice

Mice were sedated and anaesthetized in accordance with approved animal protocols at Rice University. A sharp surgical blade (15T; Sklar) was then used to cut a 0.5- to 0.75-cm midline incision through the skin and the linea alba into the abdomen. Capsule implants were expelled into the peritoneal cavity using sterile transfer pipets. The abdominal muscle was closed by suturing with 5-0 Ethicon black polydioxanone (PDS)-absorbable or other 5.0-6.0 monofilament absorbable sutures. The external skin layer was closed with PDS suture as previously described.

#### IVIS imaging

Mice were anaesthetized in accordance with approved animal protocols at Rice University and injected in the IP space with d-luciferin (300 μg/ml, 200 μl; PerkinElmer). Animals were then transferred to the IVIS manifold (IVIS Lumina K Series III, PerkinElmer) where they were kept under isoflurane anesthesia (0.25 liter/min) and maintained warm on a heated stage. Animals were imaged with an XFOV-24 lens [Field of view (FOV)-E, 12.5 mm]. Photographs and luminescent images were acquired using the Imaging Wizard feature on the Live Imaging software (PerkinElmer). Luminescent exposures were set to 15 s, with the binning set at medium, the excitation set to block, the Electron-multiplying (EM) gain set to “off” with 0-s delays between acquisitions. Animals were imaged at 5 and 10 min after d-luciferin injection. Ex vivo organs harvested from animals with IP tumors were placed on ice in PBS during collection procedures. The organs were then submerged in d-luciferin (150 μg/ml) and incubated in the dark for 4 min. Organs were then transferred onto a black sheet of paper and imaged at 5 and 10 min using FOV-C and FOV-D. All other instrument acquisition parameters were maintained constant.

#### Primate studies

Nonhuman primates (adult female Mauritian cynomolgus monkeys; age, 11 to 15 years) were purchased from DSP Research Services. All animal procedures and postoperative care were performed in accordance with the Guidelines for Care and Use of Laboratory Animals of the UIC and approved by the IACUC of UIC.

#### IP surgical implantation of capsules in NHPs

Capsule delivery was performed under standard sterile precautions in a surgical suite. The anterior abdomen was shaved and prepped from xyphoid to pubis. Under general anesthesia, a small (2 cm) supraumbilitical incision was created and a 5- to 12-mm trocar (Ethicon Endosurgery) was inserted. Pneumoperitoneum was created with CO_2_ at a pressure of 10 to 14 mmHg. After warming up with heated saline, the laparoscopic camera was inserted into the peritoneal cavity through the trocar. Under the view of laparoscopy, another small incision (1 cm) was created; an additional 5- to 12-mm trocar was inserted into peritoneal cavity for capsule administration. The capsule dose was resuspended in 50 ml of saline in a sterile bowl and collected via a 60-ml catheter tip syringe with 3 ml of airspace. A 2-ml pipette was connected by silicone tubing to the 60-ml syringe containing the capsules. The syringe loaded with capsules was inverted two to three times to ensure that the capsules were dispersed before administration. Capsules were infused into the IP cavity via the trocar incision and distributed throughout the IP cavity space. Laparoscopic videos and images were recorded. After the operation, the small incisions were sutured by layers using Vicryl 3-0 suture (Ethicon) for the muscle and Vicryl 4-0 suture (Ethicon) for the skin (subcuticular).

For delivery of capsules via the Accustick delivery system (Boston Scientific), preprocedure ultrasound evaluation of the abdomen was performed to identify an appropriate access point. An access site under which there are minimal loops of bowel and no major blood vessels was selected. The skin overlying the access site was prepped and draped according to sterile technique. A 21G needle was used to percutaneously access the peritoneal cavity under ultrasound guidance. Through this needle, a microwire (0.46 mm) was advanced into the peritoneal cavity. Over this wire, the Accustick delivery system was advanced coaxially into the peritoneal cavity. The inner metallic stylet and microcatheter was removed, leaving the outer cannula in place. Iodine-based contrast material was used in conjunction with fluoroscopy to confirm the intra-abdominal position of the Accustick. The outer cannula was connected in a sterile manner to a 60-cm^3^ luer-lock syringe, which contained the capsule dose suspended in approximately 50 ml of saline. Capsules were then delivered into the peritoneal cavity. After the operation, the small incisions were sutured by layers using Vicryl 3-0 suture (Ethicon) for the muscle and Vicryl 4-0 suture (Ethicon) for the skin (subcuticular). Healthy range for nonhuman primate red blood cell/lymphocyte count, AST, ALT, and creatinine levels was taken from the study of Xie *et al.* (*50*). All H&E imaging and pathology reports were reviewed by a board-certified veterinary pathologist at the UIC.

#### H&E staining of explanted organs and capsules

After retrieval, extracted organs or freely floating spheres were rinsed three times with PBS and fixed in 10% formalin overnight. After fixation, the spheres were rinsed twice with PBS and dehydrated in gradually ascending ethanol solutions for 20 min each time. The spheres were cleared in xylene for 10 min and incubated in a 50/50 solution of xylene and paraffin overnight at 57°C. On day 3, the spheres were transferred to paraffin twice for 1 hour each and then embedded in a paraffin mold. Subsequently, embedded spheres were sectioned at 5-μm thickness onto positively charged lysine microscope slides. Tissue sections were then stained for H&E to assess pericapsular cellular overgrowth.

### Mathematical model of IL2 pharmacokinetics

IL2 production and transport were modeled on the basis of a previous model (*40*). The IP and systemic circulation (bloodstream) were modeled as separate compartments with fluid volumes *V*_1_ and *V*_2_, respectively. The mass dynamics of IL2 in compartment *i* can be modeled by the differential equation

$$\frac{dC_{i}V_{i}}{dt}=V_{i}\frac{dC_{i}}{dt}+C_{i}\frac{dV_{i}}{dt}$$

which accounts for changes in IL2 concentration due to chemical reactions and mass transport, as well as changes in the fluid volume within each compartment.

For simplicity, we assume that fluid volumes are constant in time, which yields

$$\frac{dC_{i}}{dt}=\frac{1}{V_{i}}\frac{dC_{i}V_{i}}{dt}$$

as an expression for the compartment concentration dynamics. In general, the carrier fluid volumes used in capsule administration are much greater than the typical IP volumes *V*_1_ encountered under physiological conditions. We also note that the typical rate of IP absorption of isosmotic solutions (e.g., saline) in humans is on the order of 30 ml/hour (*66*), and thus, the typical administration volume of 50 ml would be absorbed within 2 hours after administration. Therefore, unless otherwise noted, we neglect the effects of the administration fluid volume in our simulations.

Our model assumes that IL2 is produced exclusively within the IP space from *N* implanted capsules (i.e., ignoring the physiological production of IL2 by other cells due to immune activation). The IP and bloodstream compartments are additionally linked (via lymphatic flows, diffusion, etc.), which allows for intercompartment transport of IL2.

The overall IL2 dynamics are represented by a system of differential equations

$$\frac{dC_{1}}{dt}=\frac{k_{\text{prod}}N(t)}{V_{1}}-k_{\text{trans}}(C_{1}-C_{2})$$

$$\frac{dC_{2}}{dt}=k_{\text{trans}}\frac{V_{1}}{V_{2}}(C_{1}-C_{2})-\frac{k_{\text{clr}}C_{2}}{V_{2}}$$

where *N*(*t*) represents the number of viable, IL2-producing capsules remaining at time *t* after implantation. The capsule decay due to the FBR is modeled as a simple exponential decay of the form

$$N(t)=N_{0}e^{-\lambda t}$$

where *N*_0_ represents the number of initially implanted capsules.

Relevant compartment volumes were obtained for each species by examining the literature. In mice, the blood volume was calculated to be approximately 1.2 ml on the basis of the typical mouse mass (*67*); the typical mouse IP fluid volume was found to be approximately 1 ml during IP fluid sample collection. The human compartment volumes *V*_1_ and *V*_2_ were estimated from literature sources (*43*, *68*) to be approximately 20 and 5320 ml, respectively. The NHP blood volume was estimated from the mass of each animal using the relation

$$V_{2}=65m$$

where *m* is the mass in kilograms (*46*). We were unable to find a suitable literature reference for the typical IP fluid volume in NHP, so we estimated *V*_1_ assuming that the ratio *V*_1_:*V*_2_ is the same as in human. Unless otherwise noted, model simulation was performed with the ODE15s solver in MATLAB.

### Particle swarm optimization and model fitting

The model was fitted to experimental data using the particleswarm function within MATLAB R2020a, which implements the particle swarm optimization algorithm. Unless otherwise noted, default settings were used in generating each fit.

The fit quality of each parameter set was evaluated with the normalized mean squared error (MSE) objective function

$$\text{MSE}=\sum_{i}\sum_{j}\frac{{\left(\mu_{\mathrm{ij}}-P_{\mathrm{ij}}\right)}^{2}}{\mu_{\mathrm{ij}}^{2}+\mu_{0}^{2}}$$

where μ*_ij_* is the mean IL2 concentration measured in compartment *i* at time point *j* and *P_ij_* is the predicted concentration. The normalization factor $\mu_{\mathrm{ij}}^{2}+\mu_{0}^{2}$ adjusts the relative weighting of each point in the dataset such that points with large differences in measured concentration are weighted approximately equally. We set μ_0_ = 1 pg/ml to ensure that data points below the detection limit, i.e., with measured IL2 concentrations equal to zero, would nevertheless contribute a finite weight to the objective function.

The objective function was also modified to account for the limit of detection (LOD) of the IL2 concentration assay, which was approximately 30 pg/ml for the assay used. For measured data points μ*_ij_* falling below the LOD, the penalty is only applied when the predicted concentration *P_ij_* is above the detection limit. In other words, when both data and model predict the concentrations below LOD, no penalty is applied. We used literature estimates to determine reasonable bounds for model parameters when fitting to the mouse and NHP data.

For the mouse fits, we estimated that the production rate was in the range of ~5000 to ~10,000 pg/day per capsule based on in vitro data (Fig. 1C) and thus used a wide range of ~5 × 10^3^ to 2 × 10^4^ to constrain the production rate during fitting; the wider range was to account for any differences in production efficiency in an in vivo environment. Experimental measurements of the IL2 renal clearance rate in rats (*33*) were used to estimate reasonable bounds for the mouse renal clearance, and as a result, a range of ~250 to ~750 ml/min was used for fitting. In vivo–explanted capsules (Fig. 1J) began displaying PFO as earlier as 4 days after implantation, which suggested relatively fast capsule decay dynamics. We nevertheless used a relatively wide range of 10^−2^ to 10 day^−1^ to constrain the capsule decay rate, expecting that the correct decay value will be constrained by the fitting procedure. Last, we used a wide range of 1 × 10^−4^ to 1 × 10^2^ day^−1^ to constrain the IL2 intercompartment transport rate due to a lack of literature estimates.

Similar bounds for the IL2 production rate and capsule decay rate were used for fits to the NHP data. The IL2 renal clearance rate is known to vary with animal mass; we used the postulated linear scaling of the renal clearance rate with the mass of the animal (*45*) to determine reasonable clearance rates for animals between 2 and 8 kg in mass. These values were used to construct lower and upper bounds of 7200 to 2.88 × 10^4^ ml/day to constrain the renal clearance rate in the fitting. Last, we used estimates of the IL2 intercompartmental transport rate in humans from the data of Edwards *et al.* (*34*) to determine appropriate bounds for *k*_trans_; we ultimately used lower and upper bounds of 0.1 to 24 day^−1^ to constrain the intercompartmental transport rate in NHP.

### Dose schedule optimization

A specialized solver was implemented in MATLAB to allow model simulations with multiple capsule dosing events within the simulation time span, such as in fig. S15. Given a simulation time span of length *T* and an interval between subsequent dosing events *I*, we divide the simulation into *n* discrete time blocks of length

τ=T mod I

with the size of the final block *n +* 1 equal to the remainder *T* − *n*τ.

Each time block is successively integrated using a built-in solver, with the initial conditions of each block set on the basis of the final state of the immediately preceding block. To simulate redosing, the full capsule dose is added to the remaining capsules from the preceding block to calculate the new *N*_0_.

The interval *I* between dosing events was optimized to maintain the IP concentration above an arbitrary target threshold by minimizing the scoring function

$$S={\left(C_{\text{tgt}}-C_{1,\text{min}}\right)}^{2}$$

where *C*_tgt_ is the target concentration and *C*_1,min_ is the minimum IP concentration after the initial transient. The fminbnd function within MATLAB was used for optimization.

### Estimating human intercompartmental transport rate

Human clinical trial data from a continuous IP IL2 infusion study (*34*) were used to estimate the intercompartmental transport rate (*k*_trans_) in human. The published data were digitized from figures using Graph Grabber 2.0.2 (Quintessa).

A modified version of the IL2 PK model was fitted to the clinical data using the fmincon optimizer in MATLAB; instead of IL2 production from capsules, the modified model assumes constant influx of IL2 with a fixed infusion rate. The modified model is represented by a set of differential equations

$$\frac{dC_{1}}{dt}=\frac{k_{\text{in}}}{V_{1}}-k_{\text{trans}}(C_{1}-C_{2})$$

$$\frac{dC_{2}}{dt}=k_{\text{trans}}\frac{V_{1}}{V_{2}}(C_{1}-C_{2})-\frac{k_{\text{clr}}C_{2}}{V_{2}}$$

where the parameter *k*_in_ represents the IL2 infusion rate. The infusion rate *k*_in_ was determined using the dosage administered during the trial, which was patient specific and proportional to the peritoneal surface area. Because of the unavailability of patient-level data, we estimated *k*_in_ using the typical peritoneal surface area in humans, which is approximately 1.75 m^2^ (*65*). With a daily dosage of 0.33 mg/m^2^ of peritoneal surface area per day, the infusion rate is approximately 0.57 mg/day.

The human compartment volumes *V*_1_ and *V*_2_ were estimated from literature sources (*43*, *68*) to be approximately 20 and 5320 ml, respectively. In addition, the IL2 renal clearance rate in humans was obtained from the study of Konrad *et al.* (*44*) and is approximately 1.68 × 10^5^ ml/day.

### Statistics

All statistical analyses were conducted using GraphPad Prism 8. Normality of datasets was assessed using the Shapiro-Wilks test to determine Gaussian distribution of collected data points. Two-way analysis of variance (ANOVA) and one-way ANOVA with the Holm-Sidak multiple comparisons methods were used to determine *P* values for IVIS imaging datasets. One-way ANOVA with the Holm-Sidak multiple comparisons methods were used to determine *P* values for FACS datasets. ELISA datasets were analyzed with multiple *t* tests, assuming that all sampled populations contained the same scatter. *P* values were determined using a Holm-Sidak method correction to the *t* test, with an a set to 0.05. Where indicated, ELISA datasets were analyzed using parametric unpaired *t* tests. T cell proliferation assay datasets were analyzed using a two-way ANOVA multiple comparisons method. Animal survival studies across all models were analyzed using the log-rank Mantel-Cox test. Reported colorectal tumor masses not normalized to animal body weights were tested on the basis of the assumptions of nonparametric data; a Brown-Forsythe and Welch ANOVA test was used with a method for multiple comparisons using Dunnett’s T3 test (*n* < 50). Outliers in replicates were identified using Grubb’s tests and accounted for in SEMs or SDs when included. Unless otherwise indicated as a replicate measurement, data were taken from distinct samples.

## Notes

View/request a protocol for this paper from *Bio-protocol*.
