# Supplementary material for: Clinically translatable cytokine delivery platform for eradication of intraperitoneal tumors
Source: Sci Adv. 2022 Mar 2;8(9):eabm1032. doi: 10.1126/sciadv.abm1032 (PMC8890714; doi:10.1126/sciadv.abm1032)
Supplement: Supplementary file 1 — Figs. S1 to S17 Tables S1 to S13 [file sciadv.abm1032_sm.pdf]

Science Advances
AAAS

Supplementary Materials for

Clinically translatable cytokine delivery platform for eradication of intraperitoneal tumors

Amanda M. Nash, Maria I. Jarvis, Samira Aghlara-Fotovat, Sudip Mukherjee, Andrea Hernandez, Andrew D. Hecht, Peter D. Rios, Sofia Ghani, Ira Joshi, Douglas Isa, Yufei Cui, Shirin Nouraein, Jared Z. Lee, Chunyu Xu, David Y. Zhang, Rahul A. Sheth, Weiyi Peng, Jose Oberholzer, Oleg A. Igoshin, Amir A. Jazaeri, Omid Veiseh*

*Corresponding author. Email: omid.veiseh@rice.edu

Published 2 March 2022, *Sci. Adv.* **8**, eabm1032 (2022)
DOI: 10.1126/sciadv.abm1032

This PDF file includes:

Figs. S1 to S17
Tables S1 to S13

Supplementary Tables

Table S1. Mouse antibody panel used for rechallenge T cell analysis via flow cytometry

Surface	Markers	Fluorophore	Catalog No.
Yes	CD4	PB	48-0042-82
Yes	CD8	PE-Cy7	100722
Yes	CD44	AF488	103016
Yes	CD3	PE	50-0031-U100

Table S2. Mouse antibody panel used for T cell analysis via flow cytometry

Surface	Markers	Fluorophore	Catalog No.
Yes	CD4	PB	75-0042-U100
Yes	CD8	PE-Cy7	60-0081-U100
Yes	CD25	PerCP	65-0251-U100
No	FOXp3	PE	12-5773-82
No	Ki67	FITC	11-5698-82
No	p-STAT5	APC	612599

Table S3. Mouse antibody panel used for immune cell profiling via flow cytometry

Surface	Markers	Fluorophore	Catalog No.
Yes	CD4	PB	75-0042-U100
Yes	CD8	FITC	60-0081-U100
Yes	CD19	PerCP	65-0193-U100
Yes	NK1.1	APC	25-5941-U100
No	Ki67	PE-Cy7	11-5698-82

Table S4. Mouse antibody panel used for memory T cell analysis via flow cytometry

Surface	Markers	Fluorophore	Catalog No.
Yes	CD4	PB	75-0042-U100
Yes	CD8	PE-Cy7	60-0081-U100
Yes	CD62L	PerCP	50-0621-U100
Yes	CD44	FITC	35-0441-U500

Table S5. NHP Antibody Panel used for T cell analysis via flow cytometry

Surface	Markers	Fluorophore	Catalog No.
Yes	CD4	APC	566915
Yes	CD8	PB	558207
Yes	CD25	PE	557741
No	Ki67	FITC	561165

Table S6. Summary of model parameter values

Parameter		Parameter Set						Description
Name	Units	Mouse	NHP-1	NHP-2	NHP-3	NHP-Prediction	Human	
k_{prod}	pg/day	7930.8	$2.05x10^4$	$2.05x10^4$	$2.05x10^4$	7930.8	7930.8	Per-capsule IL2 production rate
k_{clear}	mL/day	655.53	$2.33x10^4$	$2.33x10^4$	$2.33x10^4$	12240	$1.68x10^5$	IL2 renal clearance rate
k_{trans}	1/day	1.31	1.47	1.47	1.47	0.73	0.73	IL2 compartment transport rate
λ	1/day	0.91	1.47	1.47	1.47	0.91	0.91	Capsule degradation rate
V_1	mL	1.0	0.93	0.95	0.83	0.8	20	IP fluid volume
V_2	mL	1.2	247	253.5	221	220	5320	Blood volume
N_0	#	200	502	1030	1795	1800	5000	Initial capsule dose
mass	kg	N/A	3.8	3.9	3.4	3.4	N/A	Animal mass

Table S7. NHP Hematological Data at Day 7 and Day 28 for primates given various RPE-hIL2 doses

Hematological Data at Day 7			Hematological Data at Day 28	
	RBC	Lymph	RBC	Lymph
Units	x10^6/uL	x10^3/uL	x10^6/uL	x10^3/uL
Healthy Range	4.36-6.28	1.15-8.75	4.36-6.28	1.15-8.75
RPE control – 500 capsules	4.92	0.75	5.6	0.81
18.9 ug/day	4.98	4.39	5.18	1.96
35.7 ug/day	3.38	3.3	5.13	2.22
62.3 ug/day	4.78	2.93	5.18	1.27

Table S8. NHP Biochemical Data at Day 7 for primates given various RPE-hIL2 doses

Biochemical Data at Day 7					
	AST	ALT	BUN	CRE	K
Units	IU/L	IU/L	mg/dL	mg/dL	mmol/L
Healthy Range	5.67-78.75	12.00-106.89	7-21	0.59-0.95	3.8-6.4
RPE control – 500 capsules	35	11	14	0.787	6
18.9 ug/day	19	48	18	0.8188	4.1
35.7 ug/day	38	44	11	0.8009	3.8
62.3 ug/day	31	33	14	0.6857	3.6

Table S9. NHP Temperature and body weight at Day 7 and Day 28 for primates given various RPE-hIL2 doses

Temp and Weight at Day 7			Temp and Weight at Day 28	
	Temperature	Body weight	Temperature	Body weight
Units	C	kg	C	kg
RPE control – 500 capsules	37.1	4.2	35.7	4.1
18.9 ug/day	37.2	3.75	36.5	3.7
35.7 ug/day	38.6	3.8	36.5	3.6
62.3 ug/day	36.7	3.9	35.7	3.8

Table S10. NHP Biochemical Data at Day 28 for primates given various RPE-hIL2 doses

Biochemical Data at Day 28					
	AST	ALT	BUN	CRE	K
Units	IU/L	IU/L	mg/dL	mg/dL	mmol/L
Healthy Range	5.67-78.75	12.00-106.89	7-21	0.59-0.95	3.8-6.4
RPE control – 500 capsules	27	14	11	0.9396	3.8
18.9 ug/day	26	49	7	0.8397	4.1
35.7 ug/day	25	18	13	0.9563	3.7
62.3 ug/day	29	51	10	0.6788	3.2

Table S11. NHP Hematological Data at Day 7 for primates given RPE-hIL2 dose of 35.7 µg

Hematological Data at Day 7				
	RBC	WBC	Neut	Lymph
Units	x10^6/uL	x10^3/uL	x10^3/uL	x10^3/uL
Healthy Range	4.36-6.28	6.9 - 19.0	2.8 - 12.1	1.15-8.75
35.7 ug/day A	5.1	14.18	7.55	2.59
35.7 ug/day B	5.04	15.77	7.45	7.16
35.7 ug/day C	5.11	15.24	11.6	2.63

Table S12. NHP Hematological Data at Day 28 for primates given RPE-hIL2 dose of 35.7 µg

Hematological Data at Day 28				
	RBC	WBC	Neut	Lymph
Units	x10^6/uL	x10^3/uL	x10^3/uL	x10^3/uL
Healthy Range	4.36-6.28	6.9 - 19.0	2.8 - 12.1	1.15-8.75
35.7 ug/day A	5.26	3.23	1.17	1.28
35.7 ug/day B	5.51	6.44	1.45	4.01
35.7 ug/day C	5.57	4.86	3.51	1.12

Table S13. Comparison of common cytokine delivery methods

		EFFICACY						SAFETY		
Product	Technology approach	Complete efficacy as a monotherapy: Preclinical	Efficacy as a monotherapy: Clinical	Enhanced potency	High cytokine concentration at tumor site	Extended half-life	Re-dosing capability	Controlled dosing and duration of activity	Low systemic cytokine exposure	Limited toxicity
RPE-mIL2	Cytokine factory	✓	ND	✓	✓	✓	✓	✓	✓	✓
Proleukin (Novartis)	Recombinant protein	✓	✓	✗	✗	✗	✓	✓	✗	✗
NKTR-214 (Nektar)	PEGylated protein	✗	✗	✗	✗	✓	✓	✓	✓	✓
ALKS 4230 (Alkermes)	Fusion protein	✓	✗	✓	✗	✗	✓	✓	✗	✓
CEA-IL2v (Roche)	Antibody-based platform	✗	✗	✗	✓	✓	✗	✓	✓	✗
BAY 50-4798 (Bayer)	Modified protein	✗	✗	✗	✗	✗	✓	✓	✓	✓
BNT151 (BioNTech)	mRNA-based platform	✗	ND	✓	✗	✓	✓	✗	✗	✓
Ad-RTA-hIL-12 (Ziopharm)	Adenovirus-based platform	✗	✗	✓	✗	✓	✗	✗	✓	✗
NL-201 (Neoleukin)	De novo designed protein	✗	✗	✓	✗	✗	✓	✓	✓	✓

Supplementary Figures

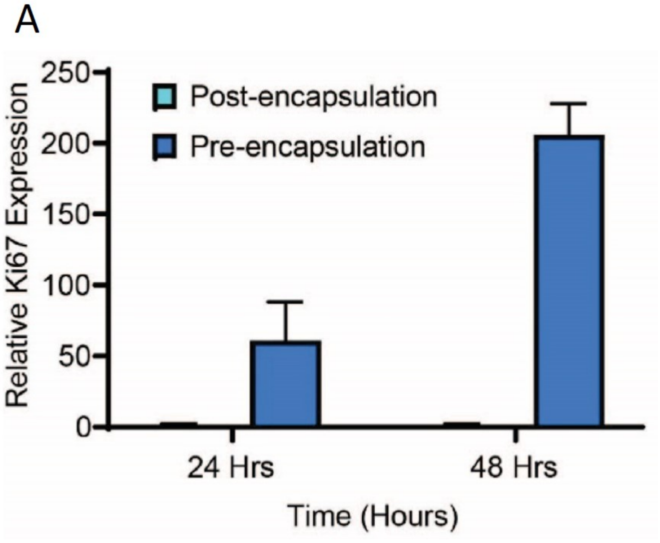

Supplementary Figure 1. 3D Cell Proliferation A) Two-Step RT-qPCR of pre-encapsulated RPE-mIL2 and post-encapsulated RPE-mIL2 cells (n=2) were plotted as relative transcript expression for Ki67 normalized to housekeeping gene GAPDH transcripts as a function of time (t=24 and 48 hours)

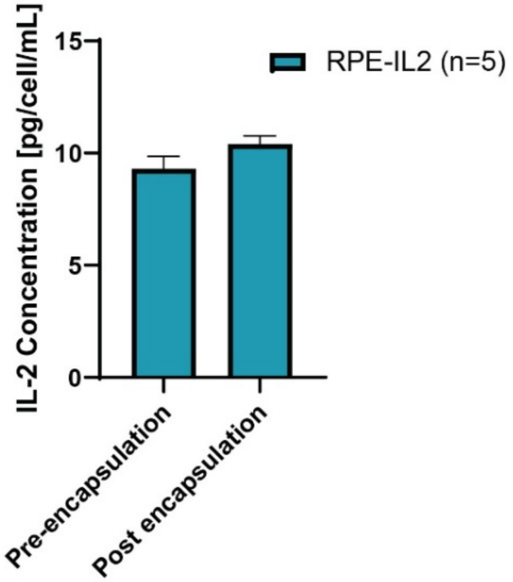

Supplementary Figure 2. 2D & 3D Protein Production. RPE-mIL2 cells were measured for mIL2 production before and after encapsulation. Values were plotted as Mean +/- SEM (n=5 capsules per measurement). mIL2 concentration from ELISA values were converted to pg/cell/mL by normalization to total cell counts within plates or capsules.

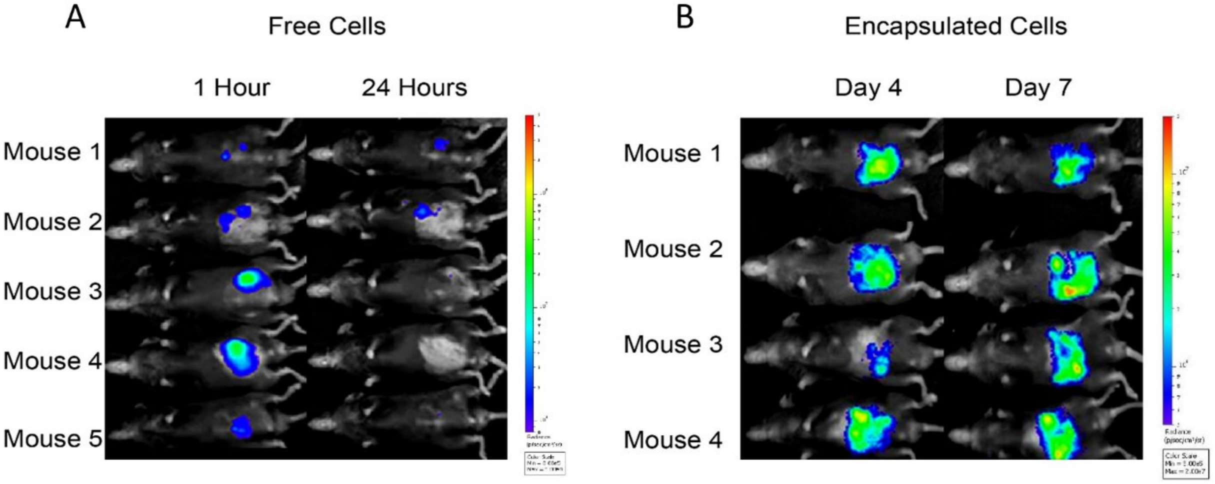

Supplementary Figure 3. Cell Viability *In Vivo*. A) RPE-Fluc cells ($4x10^6$ cells, n=5) suspended in 1x PBS were injected into the IP space of C57BL/6 mice and imaged 1 hour post injection and 24 hours post injections. Image maxima and minima of average radiance (photons/sec/cm^2/ser) scale bar displayed were $5x10^5$ and $5x10^8$ respectively. Luminescence of RPE-Fluc cells from B) 1.5 mm Core Shell Capsules (40,000 cells/capsule, 100 capsules, n=4) imaged at Day 4 and Day 7 post implantation in C57BL/6 mice. Image maxima and minima of average radiance (photons/sec/cm2/ser) scale bar displayed were $5x10^5$ and $2x10^7$ respectively. IVIS images for both panels were taken at an f-stop of 1.2, binning factor 2, FOV 24, 15 seconds exposure, and with smoothing of 3x3.

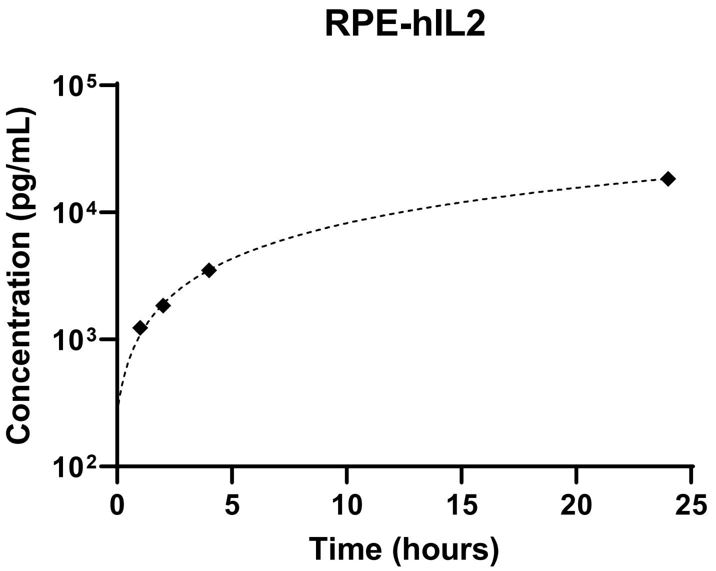

Supplementary Figure 4. hIL2 In Vitro Pharmacokinetic Data. Post-encapsulation ELISA measurements of RPE-hIL2 (pg/mL, n=4, mean +/- SEM) from core-shell capsules after 1, 2, 4 and 24-hours.

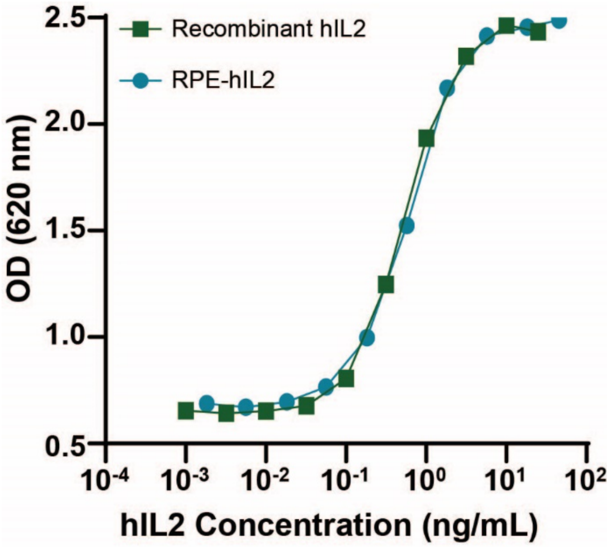

Supplementary Figure 5. RPE-hIL2 binding kinetics. Binding kinetics of clinical grade recombinant hIL2 versus RPE-hIL2 at concentrations between .001-100 ng/mL for 72 hours (n=5-6).

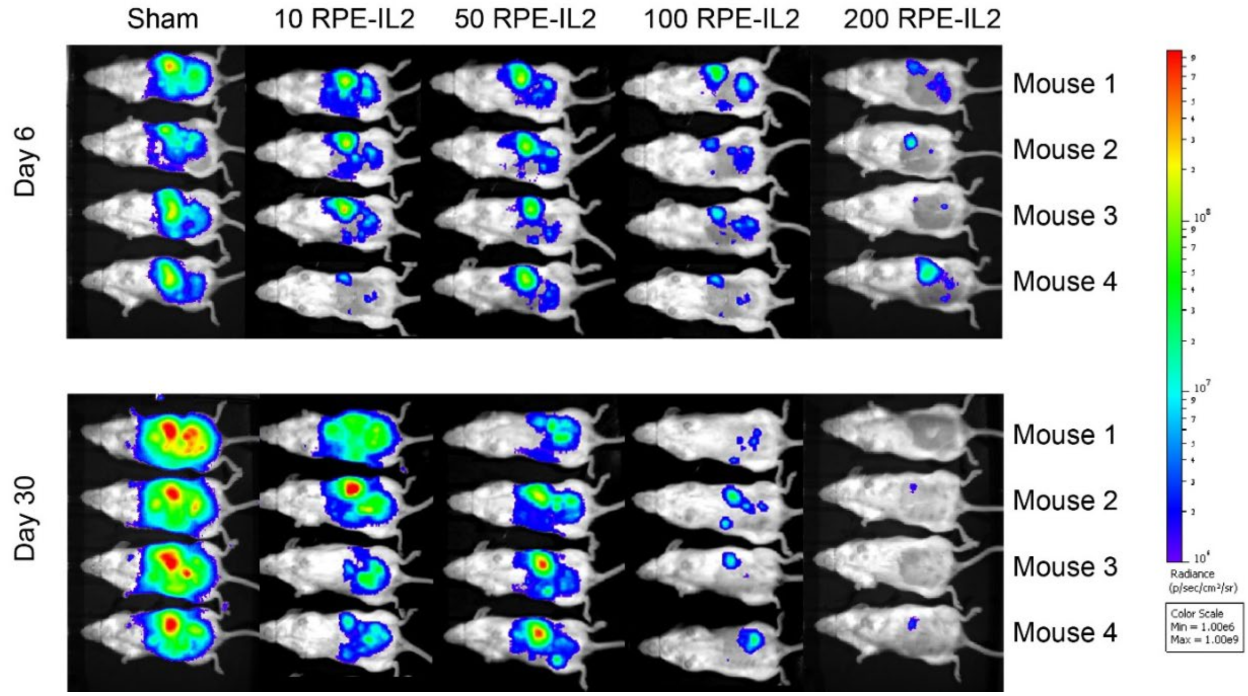

Supplementary Figure 6. Capsule Dose Study IVIS Images. Luminescence of B6 albino ID8-Fluc tumor bearing mice (n=4, female) 6 and 30 days after implantation of RPE-mIL2 capsules at various capsule doses. IVIS images were captured using f-stop 1.2, binning factor 4, FOV 24, and 15 seconds exposure. Image maxima and minima of average radiance (photons/sec/cm^2/ser) scale bar displayed were $1x10^9$ and $1x10^6$ respectively.

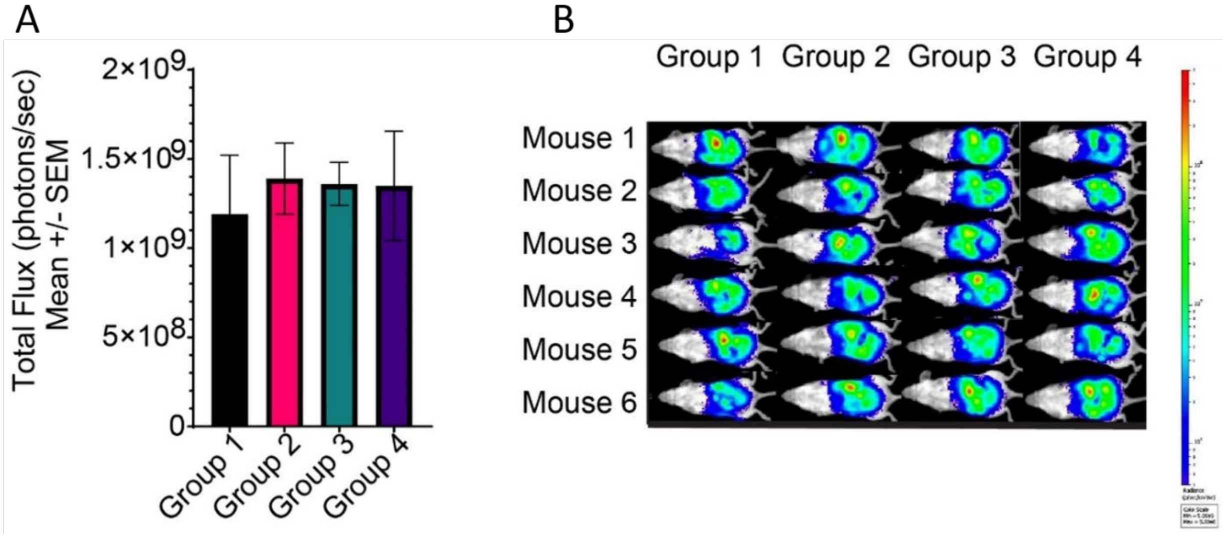

Supplementary Figure 7. ID8-Fluc B6 Albino Stratification. Group stratification images and total flux (photons/sec) for ID8-Fluc B6 albino study. A) Total flux of animals selected per cohort (n=6), no significant differences were found between stratified groups when calculated using a one-way ANOVA (alpha = 0.05). B) IVIS images showing tumor burden after stratification were acquired using an f-stop 1.2, binning factor 4, FOV 24, with 15 seconds exposure. Image maxima and minima of average radiance (photons/sec/cm^2/ser) scale bar displayed were 5x10^5 and 5x10^8 respectively.

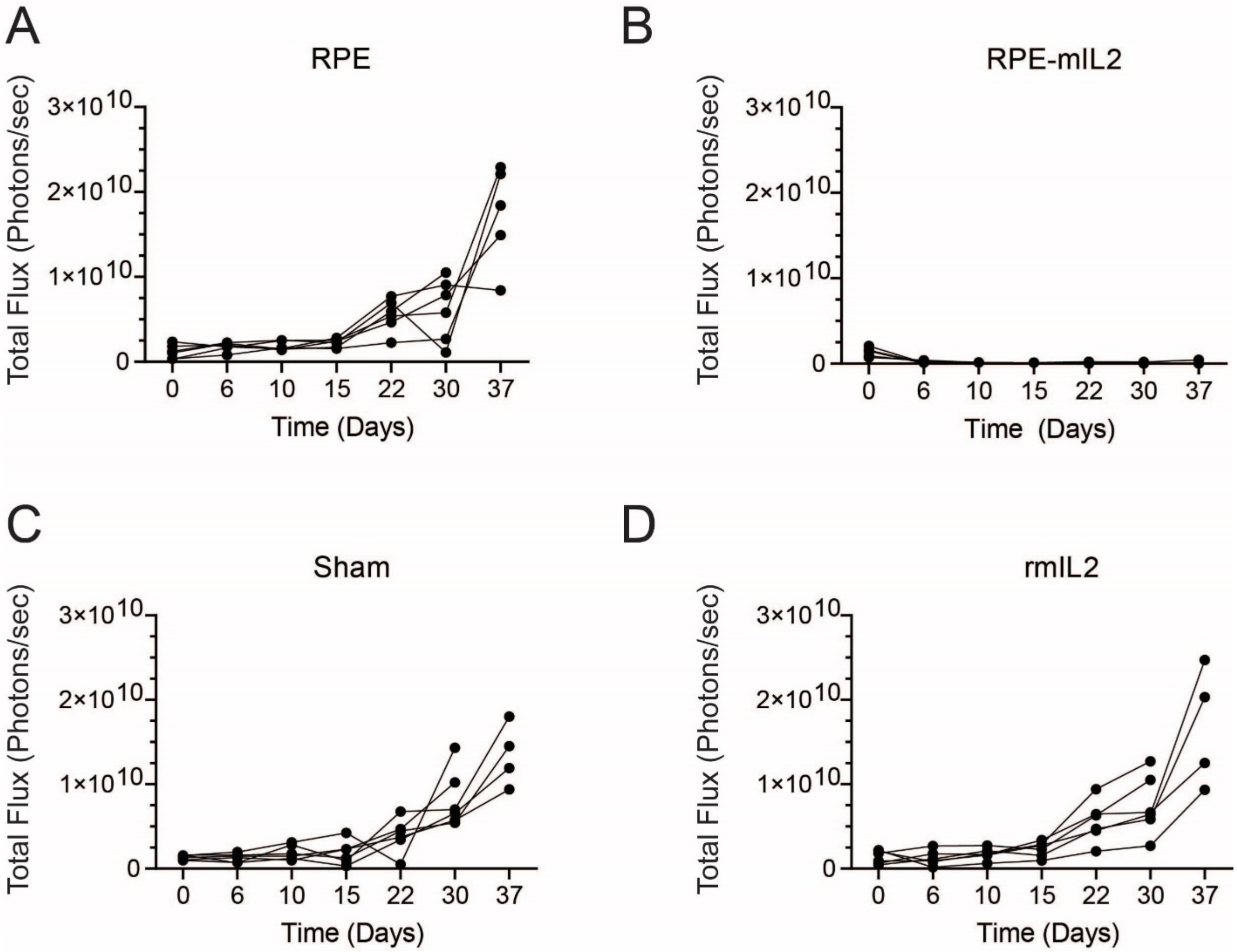

Supplementary Figure 8. ID8-Fluc Total Flux Individuals. Individual flux values for each treatment group plotted overtime for B6 albino female mice in A) RPE treatment, B) RPE-mIL2 treatment, C) Sham, or D) recombinant mIL2 injection groups from the ID8-Fluc survival study (Fig. 2C-F).

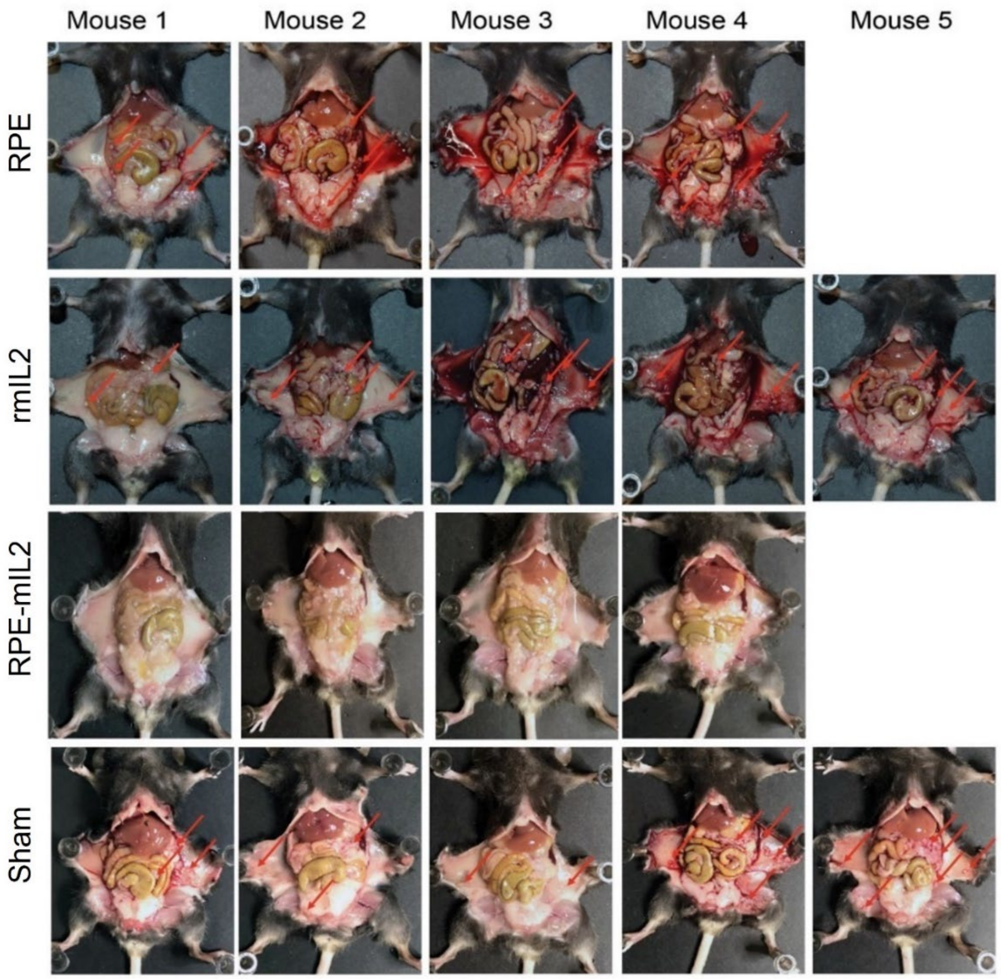

Supplementary Figure 9. ID8-Fluc Necropsy Imaging. C57BL/6 mice (n=4-5) with ovarian cancer that were treated with RPE, rmIL2, RPE-mIL2, or sham surgery were euthanized at day 75 post treatment and photographed. Red arrows indicate sites of visible tumor within the IP space. Photo Credit: Maria Ruocco, Rice University.

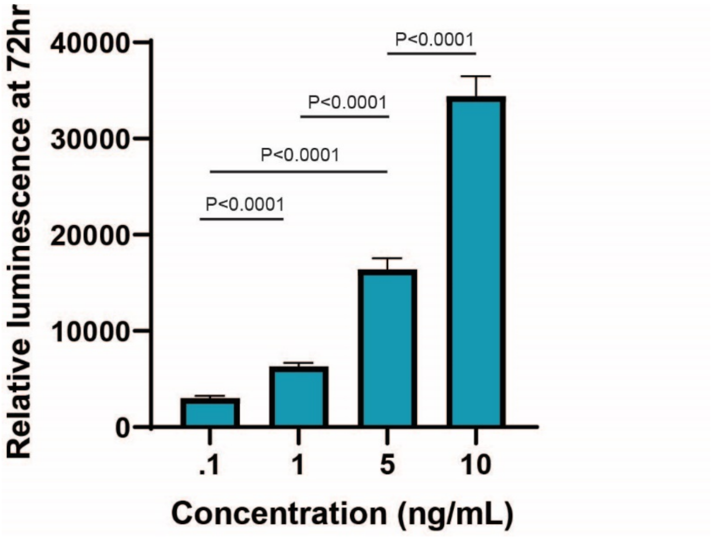

Supplementary Figure 10. Mouse T Cell Dose Response. mIL2 secreted from RPE-mIL2 capsules was administered to T Cells isolated from C57BL/6 mice spleens (n=8) at varying doses of mIL2 (ng/mL). Viability (intracellular ATP) and proliferation of T Cells was measured by luminescence (CellTiter Glo) and plotted as relative luminescence units as Mean +/- SEM. One way ANOVA was used with a Holm-Sidak's multiple comparisons test to determine significant differences between concentrations of mIL2 administered.

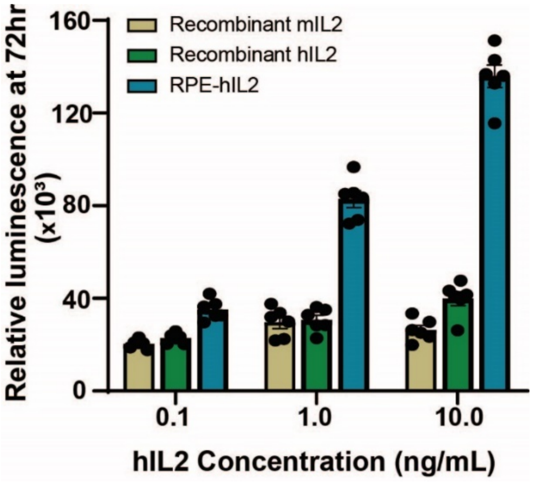

Supplementary Figure 11. Human T Cell Dose Response. Relative luminescence of human T cells measured at 72 hours (n=6) after treatment with varying doses of recombinant human IL2, recombinant mouse IL2, or hIL2 secreted from RPE-hIL2. Viability (intracellular ATP) and proliferation of T cells was measured by luminescence (CellTiter Glo) and plotted as relative luminescence units as Mean +/- SEM.

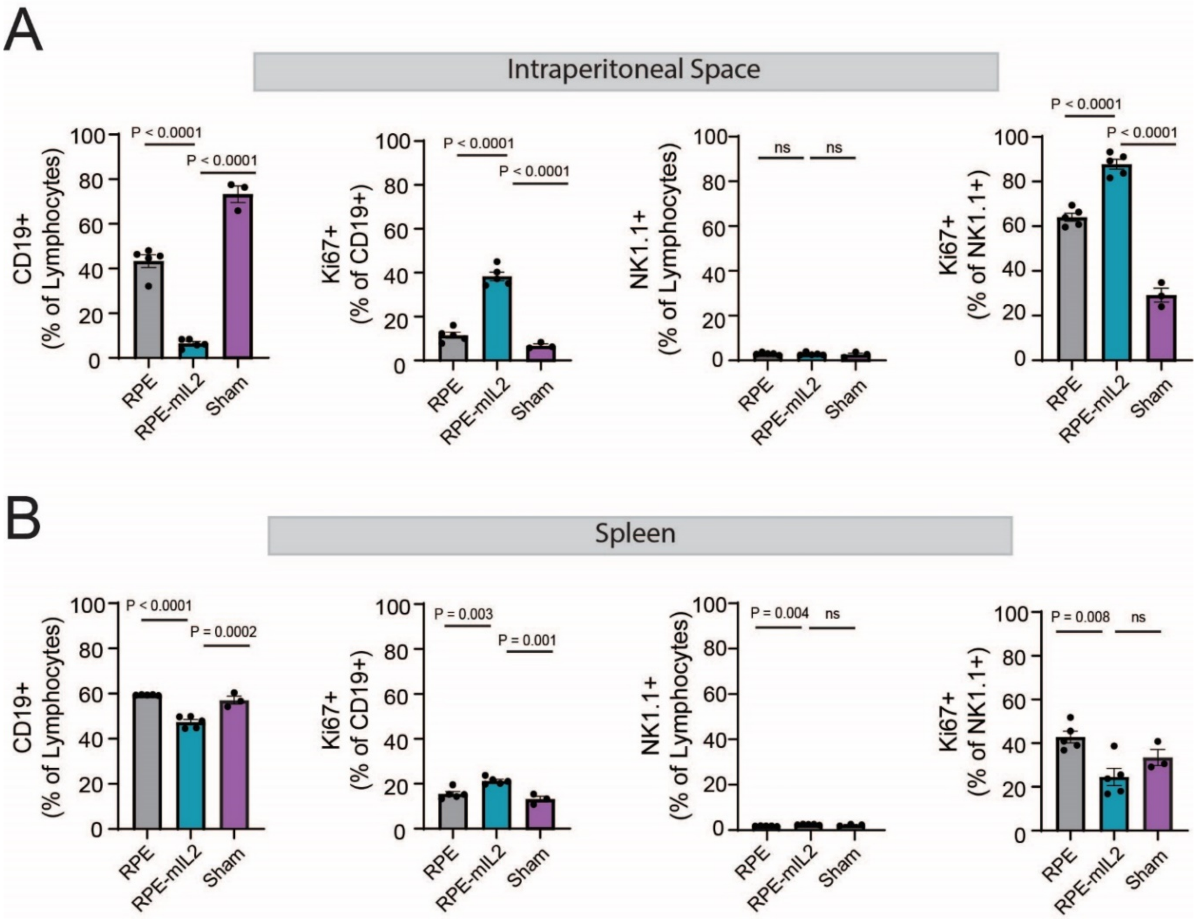

Supplementary Figure 12. Local and Systemic B and NK Cell Populations. At 7 days post treatment, mice in treatment groups (RPE vs RPE-mIL2, n=5) or control group (sham, n=3) were sacrificed and the cells of the ascites/IP fluid (local) and spleen (systemic) were stained and analyzed using flow cytometry, then plotted to show the A) local CD19+ B cells as a percentage of total lymphocytes, Ki67+, as percentage of CD19+ B cells, local NK1.1+ NK cells as a percentage of total lymphocytes, and Ki67+, as percentage of NK1.1+ NK cells. B) systemic CD19+ B cells as a percentage of total lymphocytes, Ki67+, as percentage of CD19+ B cells, systemic NK1.1+ NK cells as a percentage of total lymphocytes, and Ki67+, as percentage of NK1.1+ NK cells. One way ANOVA was used with a Holm-Sidak's multiple comparisons test to determine significant differences between the groups.

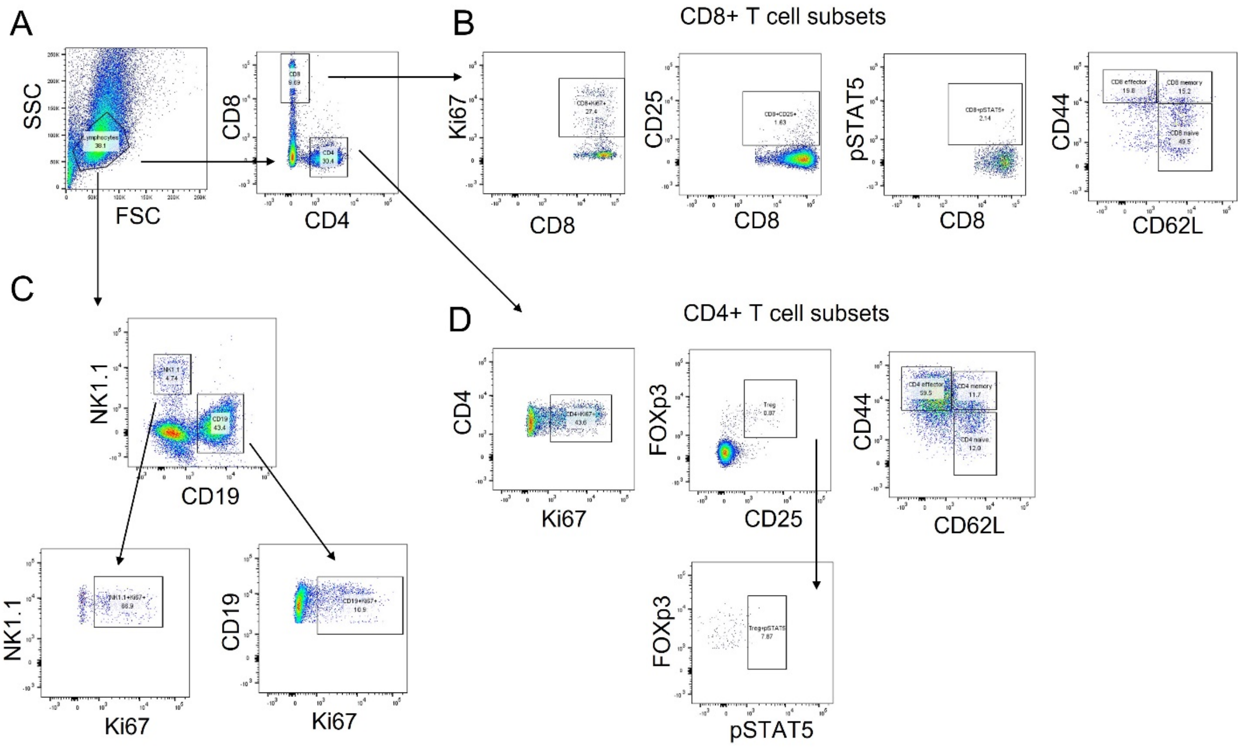

Supplementary Figure 13. Representative Dot Plots used to Gate Immune Cell Populations. ID8-Fluc tumor-bearing mice treated with RPE-mIL2, RPE, or sham surgery were euthanised at day 7 post-treatment and IP cells and spleens were processed for flow cytometry. Subpanels A-D) representative gating strategies to determine A) Lymphocytes, CD4+, CD8+ T cells, B) CD8+CD25+, CD8+pSTAT5+, or CD8+CD44+CD62L- T cell subsets, C) CD4+Ki67+, Treg, pSTAT5+Treg, or CD4+CD44+CD62L- T cell subsets, or D) NK1.1+, NK1.1+Ki67+, CD19+, or CD19+Ki67+ cells.

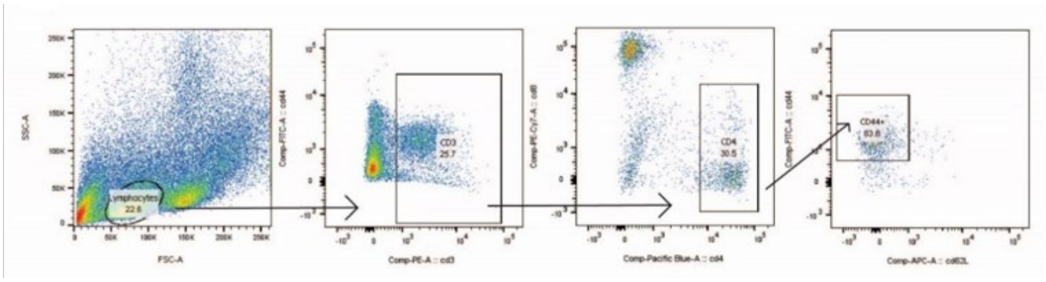

Supplementary Figure 14. Flow Cytometry Dot Plots for Memory T cells. Representative gating strategies to determine CD3+CD4+CD44+ memory T cells.

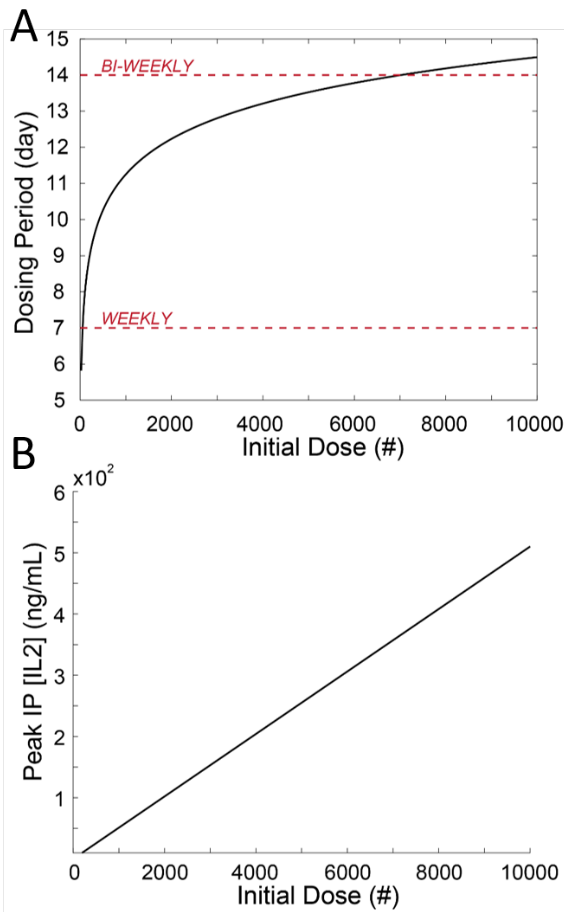

Supplementary Figure 15. Optimization of capsule administration using simple PK models A) The optimal dosing period (i.e., time between successive doses) for doses ranging from 250 to 10000 capsules; the optimal dosing period is defined such that the IP concentration never falls below the therapeutic threshold. Larger initial capsule doses can maintain therapeutic concentrations for longer periods, and thus require less frequent dosing. B) The peak IP IL2 concentration predicted for various initial doses. The peak IP concentration scales linearly with the initial capsule dose. For both (A) and (B) all parameters aside from the capsule dosage were the same as in Fig. 6e and are summarized in Supplemental Table 6 (human).

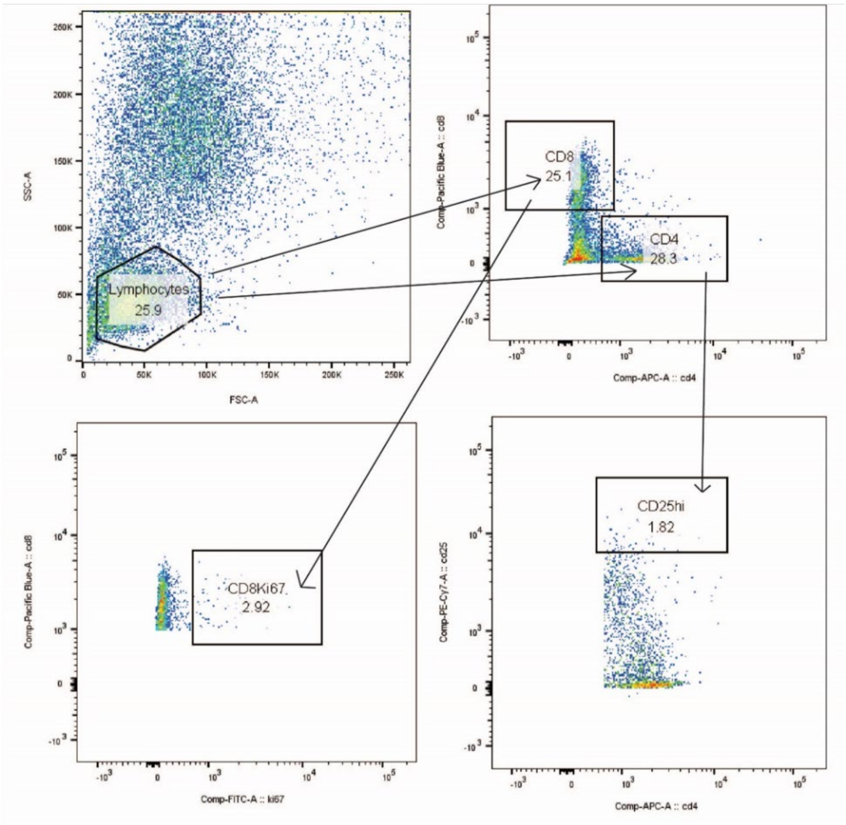

Supplementary Figure 16. NHP Flow Cytometry Dot Plots. Representative gating strategies to determine CD8+Ki67+ and CD4+CD25hi T cells.

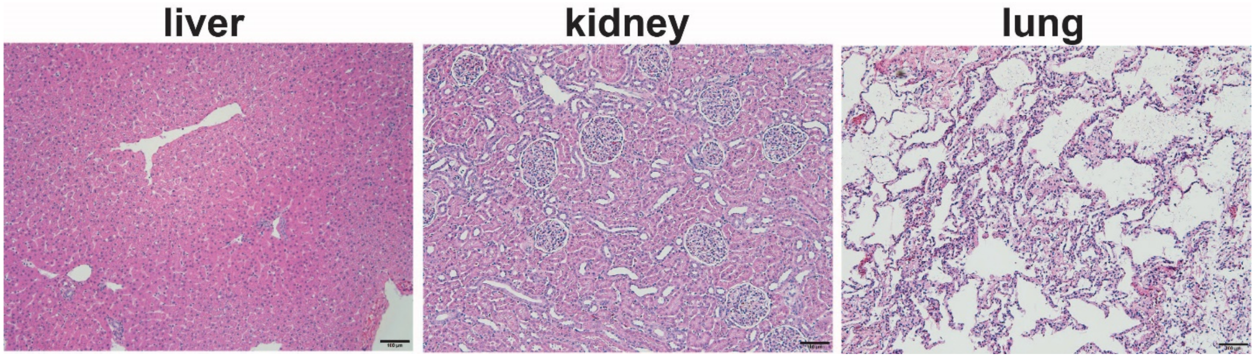

Supplementary Figure 17. H&E staining of non-human primate liver, kidney, and lung. No significant clinical abnormalities were observed during pathological analysis of organs of NHP given 62.3 µg/day of hIL2. Liver was denoted as showing centrilobular hepatocytes slightly vacuolated and determined to be mild and not clinically significant by a board certified veterinary pathologist at the University of Illinois. Lung and renal corpuscles, tubules and pelvises of left and right kidneys were unremarkable and denoted as no significant change. Scale bar is 100µm.
